# Versatile X-ray reflector extension setup for grazing-incidence experiments at SAXS facilities for liquid surface study

**DOI:** 10.1107/S1600577525003431

**Published:** 2025-06-02

**Authors:** Andrei Chumakov, Jan J. Rubeck, Matthias Schwartzkopf

**Affiliations:** a Deutsches Elektronen-Synchrotron DESY Notkestrasse 85 22607Hamburg Germany; NSRRC, Taiwan

**Keywords:** GISAXS, GTSAXS, grazing-incidence X-ray scattering, liquid interface, beam tilting, X-ray reflector, liquid surface, colloid, grazing-incidence small-angle X-ray scattering

## Abstract

Existing beamlines for *in situ* grazing-incidence small-angle scattering on liquids are either limited in angular range or incompatible with the large sample–detector distance required for submicrometre resolution. We present a low-cost, easily assembled beam-tilting extension for synchrotron-based ultra-small-angle X-ray scattering (USAXS) facilities, enabling grazing-incidence and transmitted scattering (GIUSAXS, GTUSAXS) studies on liquid surfaces. The setup is compatible with standard USAXS beamlines and requires only ∼0.5 m of additional space at the sample stage. It allows X-ray beam incidence angles of up to ∼0.6° at the liquid surface, equal to twice the angle of incidence on a reflector and below its critical angle of typical materials (*e.g.* silicon, germanium, *etc*.), and provides access to a *q*-range of approximately 0.003–0.5 nm^−1^. The system was tested at P03 beamline (DESY) using polystyrene nanoparticles (∼197 nm) self-assembled at the air/water interface. The recorded GIUSAXS and GTSAXS patterns revealed features characteristic of near-surface hexagonally ordered monolayers and multilayer assemblies, validating the system’s resolution and sensitivity. The proposed scheme enables selective depth profiling and expands the research capabilities of existing small-angle X-ray scattering synchrotron facilities for *in situ* studyies of submicrometre nanostructured objects at liquid surfaces under grazing-incidence geometry, while remaining fully compatible with complementary techniques such as grazing-incidence wide-angle scattering and total reflection X-ray fluorescence.

## Abstract 2

We present an easily assembled, low-cost beam-tilting extension for synchrotron-based ultra-small-angle X-ray scattering (USAXS) / small-angle X-ray scattering (SAXS) beamlines enabling grazing-incidence (GIUSAXS) and transmitted (GTUSAXS) experiments on liquid surfaces with negligible loss of X-ray flux. The setup is implemented at the sample stage with ∼0.5 m of additional space and provides incidence angles up to ∼0.6°, corresponding to approximately twice the critical angle of typical reflector materials.

## 1. Introduction

Many critical applications in surface designs related to biology, chemistry, medicine, nanoelectronics and materials science focus on air/liquid and liquid/liquid interfaces (Gangwar *et al.*, 2024 ▸; Fu *et al.*, 2024 ▸). The scale of the investigated objects and their agglomerates ranges from a few angstroms to a few micrometres or more. Both conventional troughs and Langmuir troughs are widely used as liquid interface holders, allowing the concentration of molecules or particles on the surface to be regulated utilizing a movable barrier. This approach allows researchers to perform *in situ* or *operando* studies of model systems at water-based interfaces under controlled conditions. Such microscopy techniques as optical microscopy, Brewster-angle microscopy, Raman microscopy, liquid-phase atomic force microscopy (AFM), *etc*., including in combination with surface pressure measurements, are widely used for *in situ* observation of liquid surfaces in laboratory settings. The disadvantages of such methods include small localization of analysis at the nanoscale, limited to a few tens of square micrometres, predominantly qualitative rather than quantitative information, and limited temporal and spatial resolution.

X-ray reflectometry, as well as wide-angle and small-angle scattering in grazing-incidence geometry mode (GISAXS, GIWAXS), provide unique information during the investigation of the internal structure and order of nano-objects on and below the surface of a sample or film under study from the entire illuminated near-surface volume (Hexemer & Müller-Buschbaum, 2015 ▸; Schwartzkopf & Roth, 2016 ▸; Steele *et al.*, 2023 ▸). Many *in situ* GISAXS observations during the self-assembly of nanoparticles are performed at fixed incident angles on solid substrates via spray deposition or printing methods (Herzog *et al.*, 2013 ▸; Cao *et al.*, 2021 ▸). In contrast to transmission X-ray beam scattering (90° incidence angle), the grazing-incidence geometry mode assumes a very small incidence angle α_i_ of the order of magnitude of one or more critical angles α_c_ of the film material under investigation and does not exceed 1°. Thus, due to the large footprint of the beam in the sample, the necessary scattering interaction between the incident wave and the investigated structural features of the sample is provided with high sample statistics. At the same time, by changing the angle of incidence from 0.8α_i_ to a factor of 3–4 of α_c_ of the material the researcher can selectively calculate the beam penetration depth from several nanometres to several micrometres, respectively (Reichert *et al.*, 2003 ▸). Knowledge of the depth of the investigated objects in the near-surface region is especially relevant when studying liquid surfaces in the *in situ* mode (Fu *et al.*, 2024 ▸).

In contrast to laboratory X-ray instruments, the main advantage of synchrotron radiation is the high degree of beam coherence, low divergence, flexibility in the choice of photon energy, and very high fluxes. This allows *in situ* and other similar time-resolved experiments with very high precision and high temporal resolution down to micro- and picoseconds with sufficiently high data statistics (Schwartzkopf *et al.*, 2021 ▸). The establishment of a synchrotron as a source of X-ray radiation for materials research is a very expensive large-scale project (Grabowski *et al.*, 2021 ▸). The division of the experimental part of the synchrotron into highly specialized high-precision research facilities (beamlines) with a specific set of techniques implies the creation of expensive unique instrumental equipment (a set of optical and measuring devices) together with radiation protection facilities (optical and experimental hutches).

For natural reasons, it is impossible to realize a controlled inclination of the liquid surface from the horizontal direction. There are known rather exotic ways to create inclined liquid surfaces at an angle of no more than 5° (Villegas *et al.*, 2019 ▸), but the majority of studies require a sufficiently thick layer of liquid in a single locally undivided volume (Yun & Bloch, 1989 ▸). The study of air/liquid and liquid/liquid interfaces requires controlled tilting of a pre-prepared horizontal synchrotron beam to a given angle (Pershan & Schlossman, 2012 ▸). For this purpose, a single- (Smilgies *et al.*, 2005 ▸) or two-crystal deflector (with different crystal orientations) using the principle of Bragg diffraction to tilt the X-ray beam (Honkimäki *et al.*, 2006 ▸; Arnold *et al.*, 2012 ▸; Murphy *et al.*, 2014 ▸; Konovalov *et al.*, 2024 ▸; Shen *et al.*, 2022 ▸) is often used. In addition, examples of an in-built optional scheme for a small fixed beam tilt using a fixed optical X-ray mirror are sometimes found (Pershan & Schlossman, 2012 ▸; Smilgies *et al.*, 2005 ▸; Jeng *et al.*, 2010 ▸). However, this possibility of using a pre-integrated mirror is very rare and is rather an additional option. These optical strategies are fundamental for configuring synchrotron beamlines to study liquid surfaces with controlled penetration depth and incidence angle. Unfortunately, to date, all realized beamlines for the study of liquid interfaces have a spatial limitation in the sample–detector distance (SDD) as well as in the size of the diffractometer itself. The SDD of such stations ranges from 2 to 4 m. The main techniques of such beamlines are predominantly X-ray reflectometry and grazing-incidence diffraction/GIWAXS with an optional limited range in the GISAXS region that does not sufficiently resolve structural features larger than 100 nm.

There is an example of the implementation of a compact ultra-small-angle X-ray scheme (Chumakov *et al.*, 2019 ▸) based on two-dimensional compound refractive lenses (Snigirev *et al.*, 1996 ▸; Schroer, 2000 ▸), which allows successful measurement of submicrometre objects at SDDs on the order of 1.5 m. However, a significant reduction in SDD also requires a corresponding significant reduction in pixel size on the measuring 2D detector to preserve spatial resolution (Chumakov *et al.*, 2019 ▸; Pauw, 2014 ▸). Most modern single-photon-counting detectors, which are commercially available and applied, have a linear pixel size of 55–172 µm due to the physical limitation on charge divergence into the chip media (Fröjdh *et al.*, 2024 ▸). This, in turn, leads to the need to significantly increase the SDD to several tens of metres to maintain high resolution when investigating submicrometre-sized structures (Narayanan *et al.*, 2022 ▸; Bras *et al.*, 2003 ▸; Pauw, 2014 ▸; Haas *et al.*, 2023 ▸; Buffet *et al.*, 2012 ▸). Thus, at the present moment researchers have an opportunity to perform GISAXS measurements at the liquid interface in beamlines with a tilted beam and relatively small SDD, as well as to study submicrometre objects with high resolution at large SDD using the SAXS and USAXS methods, but only in the transmission mode and without the possibility of beam tilting.

In this paper, we propose a low-cost, easily assembled temporary scheme for tilting the X-ray beam for grazing-incidence and transmitted USAXS (GIUSAXS and GTSAXS, respectively) to measure objects or agglomerates on a liquid surface at a controlled target angle of incidence. It can be implemented on most SAXS beamlines or facilities without loss of X-ray beam flux and requires only the order of 0.5 m of additional free space in front of the sample node. Different surfaces with minimal roughness can be used as a beam reflector (tilt), considering the footprint of the deflected X-ray beam. The maximum beam incidence angle on the liquid is limited to the value of the double critical angle of the reflector material, which in most cases covers all the basic needs of GISAXS at the liquid interface. This scheme can also be easily complemented by GIWAXS (Konovalov & Vorobiev, 2013 ▸) and total reflection X-ray fluorescence (TXRF) techniques (Novikova *et al.*, 2003 ▸; Wobrauschek, 2007 ▸; Fernández-Ruiz, 2022 ▸). The proposed scheme was tested on the behavior of a colloid of polystyrene spheres at and below the air/liquid interface. The self-organization of the investigated system near the liquid surface and in the near-surface layer was demonstrated in *in situ* experiments. The proposed scheme opens a significant and easy extension of the research capabilities of existing small-angle X-ray facilities worldwide.

## 2. Description of the proposed scheme

### 2.1. Theoretical introduction

To understand and optimize X-ray interaction with matter in the 6–25 keV energy range, particularly for grazing-incidence techniques like GISAXS and GIWAXS at liquid interfaces, it is essential to consider both the photoelectric effect and Rayleigh scattering as dominant processes (Als-Nielsen & McMorrow, 2011 ▸). The refractive index of materials for X-rays is slightly less than 1 and is expressed as *n* = 1 − δ + *i*β, where the real part δ governs refraction and scattering, and the imaginary part β relates to absorption and radiation damage. Total external reflection occurs below the critical angle α_c_ ≃ 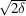, enabling surface-sensitive techniques such as GISAXS and GIWAXS. The reflectivity and penetration depth of X-rays depend on the angle of incidence and the optical properties of the medium, which can be tuned to either enhance surface sensitivity or probe deeper into the sample. Understanding these parameters is essential for optimizing measurement conditions and minimizing beam-induced damage. A more detailed theoretical and practical analysis is given in Section S1 of the supporting information (SI).

### 2.2. Practical description

The above theoretical introduction and description in Section S1 (SI) highlight two key practical considerations related to the reflector and the sample surface. For efficient GISAXS experiments, the reflector inclination angle (α_i_r_) is typically selected at about 80–85% of its critical angle (α_c_r_), maximizing reflectivity. Due to equal angles of incidence and reflection, the incidence angle on the liquid surface (α_i_s_) becomes twice that of the reflector angle (α_i_ = 2α_i_r_). Thus, even for materials of reflector and liquid with similar electron densities, this setup allows selective probing of both the air/liquid interface (for angles below α_c_) and structures beneath the surface (above α_c_).

For studies specifically targeting nanoobjects directly at the liquid interface, selecting α_i_s_ around 80% of the liquid’s critical angle is optimal, providing surface-sensitive data within a few nanometres depth. Increasing α_i_s_ above the critical angle enhances beam penetration depth significantly, enabling depth-resolved structural analysis through GIUSAXS, GIWAXS or TXRF techniques. Detailed calculations of critical angles, optimal incidence angles and reflectivities for common mirror materials, including silicon, germanium and thin metal films, are summarized in Section S2, Tables S1 and S2 (SI).

The reflector dimensions are chosen considering the beam footprint, which depends primarily on the incidence angle and the vertical beam size. Practical guidelines for calculating the required mirror dimensions, as well as considerations related to liquid surface experiments, are addressed in detail in Section S2 (SI) and the relevant literature (Pershan & Schlossman, 2012 ▸; Widom, 2004 ▸; Höfling & Dietrich, 2024 ▸; Konovalov *et al.*, 2022 ▸). In comparison with existing GISAXS setups designed for liquid interfaces, our approach offers a low-cost and mechanically simple alternative. Double-crystal deflector systems, such as those described by Honkimäki *et al.* (2006 ▸) and Jeng *et al.* (2010 ▸), allow continuous variation of the incidence angle while maintaining the beam footprint fixed on the sample, often using synchronized goniometer motions or slit assemblies. These setups are highly precise and efficient, but their integration demands complex mechanics, beamline-specific software and extended alignment time. Our single-reflector scheme sacrifices beam position invariance during angle changes, but offers a compact, transferable solution requiring only minor vertical sample adjustments. A comparison of key features is presented in Table S5 (SI).

## 3. Experimental part

### 3.1. Experimental setup

**Figure 1:**
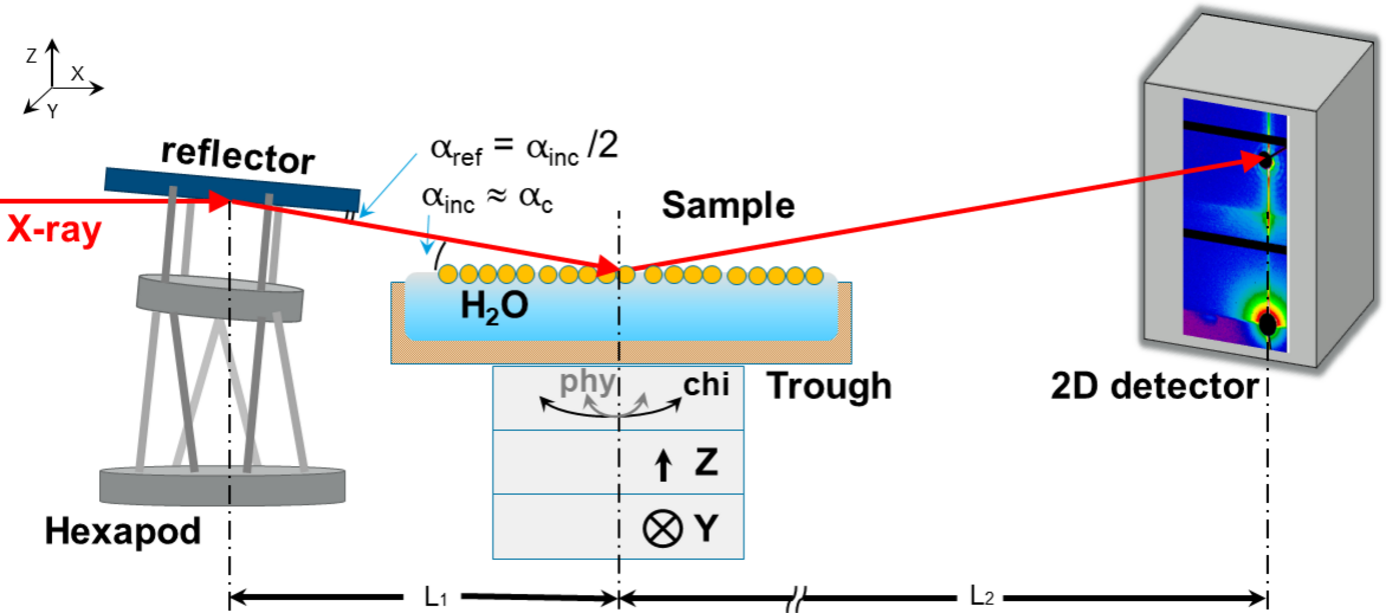
Schematic representation of the reflector and sample assembly combination for the USAXS setup. The reflector is positioned on a specialized holder mounted on a hexapod with six degrees of freedom. The Teflon liquid trough is mounted on the sample stage with the possibility of adjusting the horizontal position of the trough, as well as adjusting the air/liquid interface level in height and moving the sample perpendicular to the beam axis.

Fig. 1 ▸ schematically depicts the arrangement of the main nodes for the beam deflection experiment on the liquid surface. To organize the beam deflection scheme in the experimental hutch of the majority of the SAXS/USAXS beamline, it is necessary to allocate an additional ∼0.5 m of space *L*_1_ in front of the sample node. In this location, a motorized device with the possibility of adjusting the reflector in four degrees of freedom (vertical *Z* and horizontal *Y* displacements orthogonal to the beam, CHI and PHI tilts, in planes perpendicular and parallel to the beam axis) was installed. In our case, a hexapod with six degrees of freedom was used.

**Figure 2:**
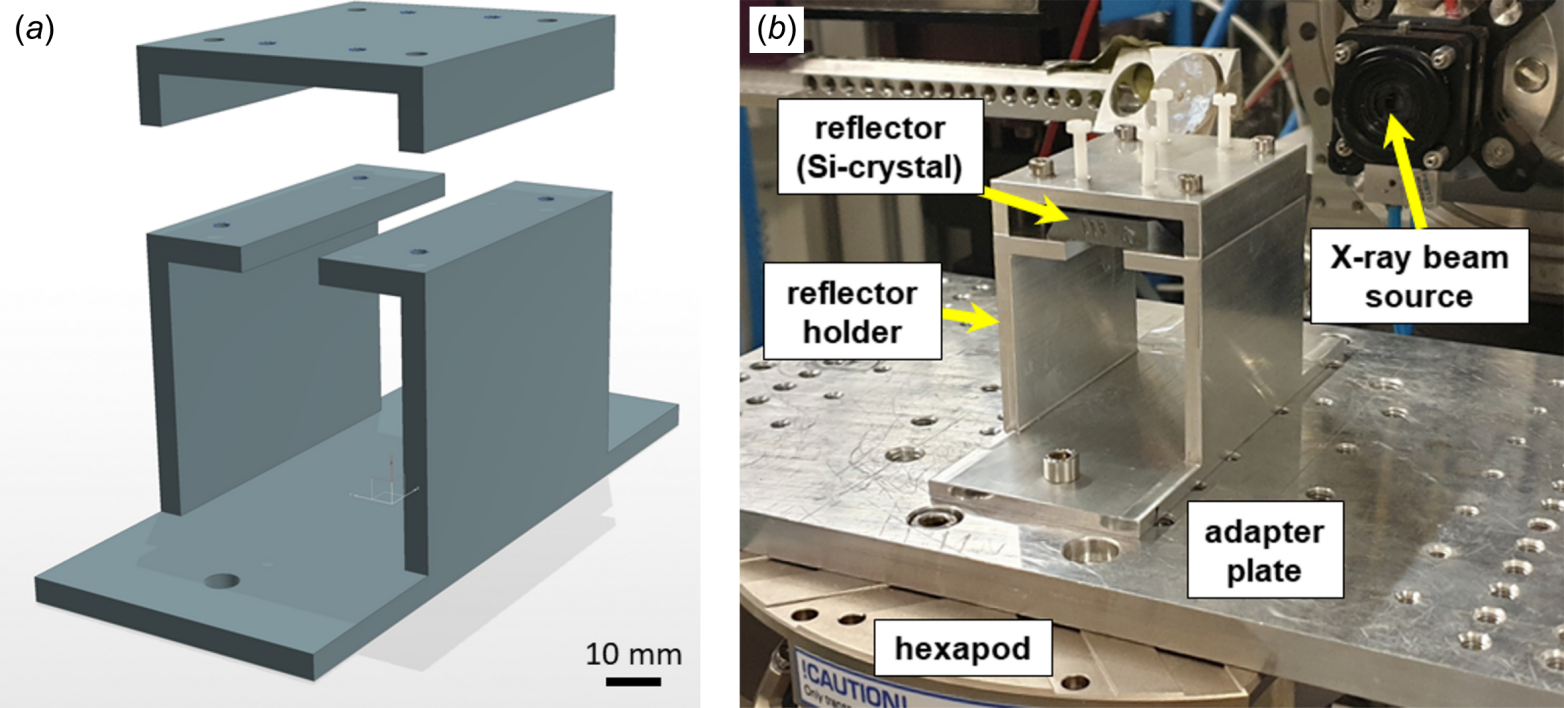
(*a*) Sketch of the reflector holder. The main (lower) part of the holder is designed for mounting the reflector plate at the top above the slot, reflective side down. The upper part is designed for soft pressing of the reflector by plastic screws to the main part of the holder. (*b*) Silicon reflector at final assembly, mounted on the hexapod in the experimental hutch.

A metal reflector holder was mounted on top of the hexapod, Fig. 2 ▸. The holder can be easily produced from a square section of aluminium tube. It consists of a main (bottom) and a hold-down (top) part. The reflector is placed over the slot on top of the main part of the holder. The reflecting plane of the reflector should be orientated downwards to deflect the X-ray beam onto the liquid surface of the sample. The length of the reflector is chosen based on the expected footprint of the beam *l*_fp_ on the reflector, the vertical size of the beam, its energy and the minimum planned angle of the reflector, according to equation (S9) in SI. The clamping part of the holder is mounted on top and is rigidly fixed with bolts in the four outer holes. Plastic bolts are screwed into the inner four holes of the clamping part for soft fixation (pressing) of the reflector to the main part of the holder, Fig. 2 ▸(*b*). Parameters of the materials used in manufacturing, as well as detailed technical drawings of the holder, are given in Table S3 and Figs. S1–S4 (SI).

**Figure 3:**
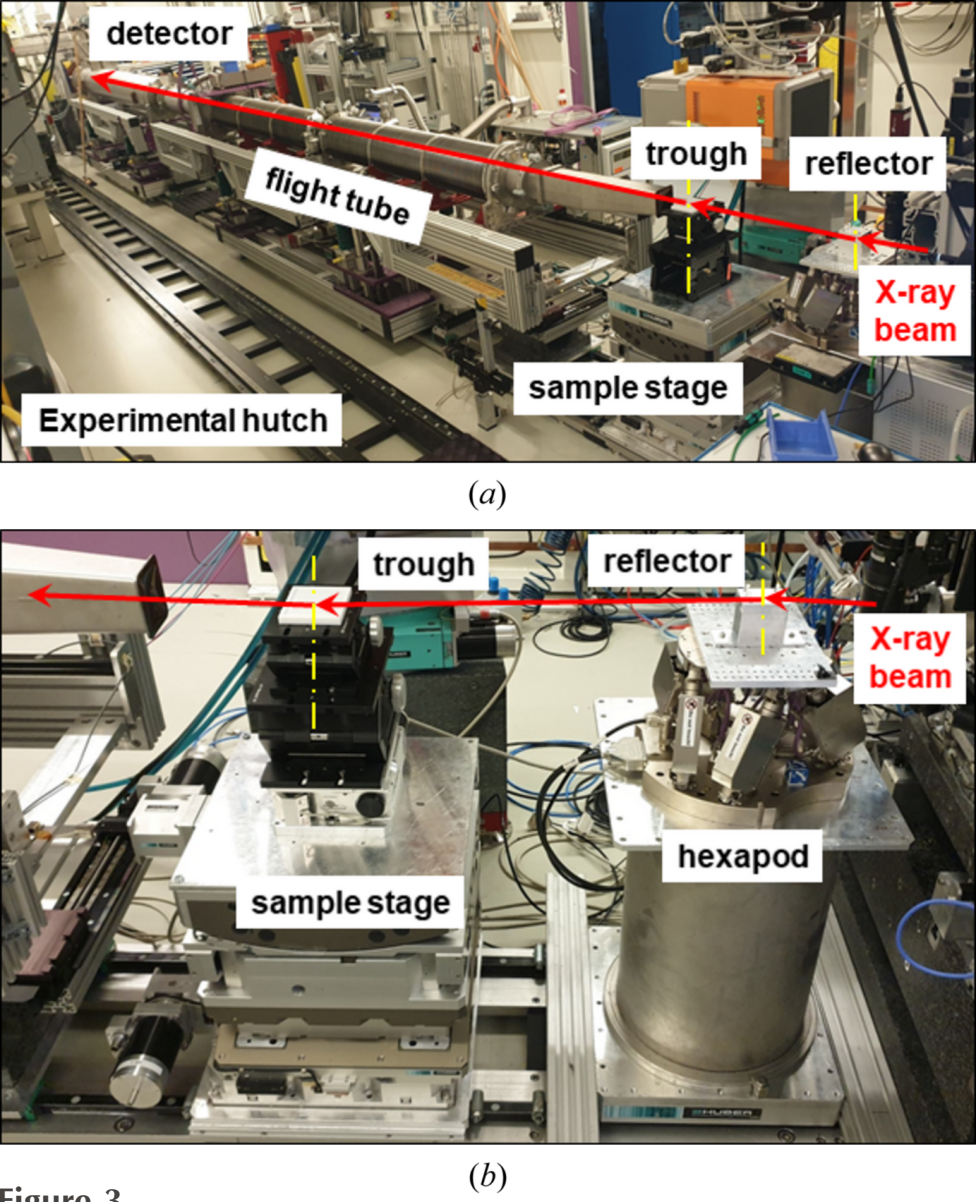
(*a*) General view of the setup with the realized scheme of tilting the X-ray beam onto a liquid surface for the USAXS study inside the experimental hutch EH1 of the P03 station at the PETRA III synchrotron (DESY, Hamburg, Germany). (*b*) The reflector assembly was placed on the hexapod and the sample assembly was placed on the Huber sample stage (side view). Propagation directions of a collimated monochromatic X-ray beam focused on the reflector. The vertical axes of the reflector and the sample are shown by yellow dashed-dotted lines.

A sampling stage (HUBER Diffraktionstechnik GmbH) with similar four degrees of freedom was positioned after the reflector assembly on the beam axis line, Fig. 3 ▸(*b*). A Teflon trough with internal dimensions of 70 mm × 90 mm was positioned slightly below the horizontal axis of the reflector so that it was possible to bring the X-ray beam to the middle of the air/water interface surface. The top plane of the trough should be adjusted horizontally. Adjusting the beam position to the center of the trough is done by moving the position of the liquid interface vertically. To preserve the quality of pure GIUSAXS signal, a vacuum tube is placed immediately after the trough, covering as much of the SDD as possible, labeled as *L*_2_. A 2D detector installed at the end of the circuit allows 2D scattering patterns from the sample at the air/liquid interface to be obtained for further analysis.

The proposed reflecting scheme was tested at the experimental hutch EH1 of beamline P03 at the synchrotron PETRA III [DESY, Hamburg, Germany (Buffet *et al.*, 2012 ▸)]. An X-ray beam with energy 11.83 keV (wavelength λ = 1.048 Å, Δλ/λ = 10^−4^) was focused on the reflector position by beryllium compound refractive lenses (CRLs) and had a size (W × H) of 25 µm × 35 µm. The reflector–sample distance *L*_1_ was 500 ± 1 mm. The SDD (*L*_2_) was 8985 ± 2 mm. Pilatus 2M (Dectris Ltd, Switzerland) with pixel size 172 µm × 172 µm and pixel array format (W × H) 1475 × 1679 pixels was used as a 2D detector for GIUSAXS mode. The reciprocal *q*-space and SDD were calculated using the Ag-behenate calibrant. The data processing were performed using the *DPDAK* software package (Benecke *et al.*, 2014 ▸).

A polished silicon crystal [Si (111) from Si-Mat], 55 mm long and 24 mm wide, was used as a test reflector. The calculated longitudinal footprint *l*_fp_ of the beam at the reflector did not exceed 34.5 mm for the minimum used reflector tilt angle α_i_r_ = α_i_s_/2 = 0.0417°. This corresponded to half of the beam incidence angle on the water surface α_i_s_ = 0.083°, which is 80% of the critical H_2_O angle α_c_H2O_ = 0.104° at the used beam energy. The beam footprint on the liquid surface for this angle was *l*_fp_s_ ≃ 17.2 mm. Increasing the angle of incidence led to a decrease in the reflector and sample footprint, according to equation (S9) of SI.

Fig. 3 ▸(*a*) shows an example of a circuit implementation with an optional reflector for deflecting a horizontal X-ray beam onto a liquid surface for grazing-incident geometry experiments (GIUSAXS, GIWAXS, TXRF) inside the experimental hutch EH1 beamline P03. The monochromatic focused X-ray beam (right) hits the pre-tuned reflector. After being deflected by a preset slip angle, the beam is redirected to the surface of the liquid sample at the resulting double angle of the reflector. The liquid is in a trough, which is fixed at the sample stage. Depending on the angle of incidence on the liquid surface, the beam either slides along it (α_i_ < α_c_) or starts to partially penetrate deep into the liquid (α_i_ > α_c_), scattering on the investigated nanostructured objects. After the trough, a vacuum flight tube is installed to avoid scattering of the useful GIUSAXS signal in the air. A 2D detector is installed at the end of the tube to record the scattered image. Fig. 3 ▸(*b*) gives a more detailed view of the location of the reflector node and the sample node at the beginning of the experimental hutch. The vertical axes of both nodes are marked with yellow dashed lines. An example of images of a polished silicon crystal, installed (*a*) and aligned (*b*) as a reflector, is also shown in Fig. S6 (SI). A red laser was used to visualize the position of the X-ray beam, with its axis coinciding with the axis of the X-ray beam. The camera lens in Fig. S6 (SI) was directed toward the X-ray beam. Also, the proposed scheme enables effortless switching between GISAXS on liquid surfaces and conventional SAXS within the same experiment by moving the reflector out of the beam path using the vertical *Z* motor and replacing the liquid trough with a standard SAXS sample. If necessary or possible, an active anti-vibration system can also be installed below the trough.

A potential limitation of the proposed setup arises from the extended air path introduced between the X-ray reflector and the liquid sample, which may increase parasitic background scattering, particularly at ultra-small angles. Although the primary beam remains collimated by upstream optics and pinholes, scattering from air and mounting structures can reduce the signal-to-noise ratio. This effect is more pronounced at lower photon energies and longer SDDs. To mitigate this, we recommend minimizing the air gap and, when feasible, using helium purging or evacuated flight tubes. While our tests showed that angular resolution remains acceptable for grazing-incidence angles in the 0.05°–0.5° range, users should ensure mechanical stability and proper shielding of the reflector environment to maintain beam quality. These considerations are particularly relevant when aiming for high contrast and sensitivity in liquid surface studies (see also Section S2 of SI).

### 3.2. Alignment procedure

**Figure 4:**
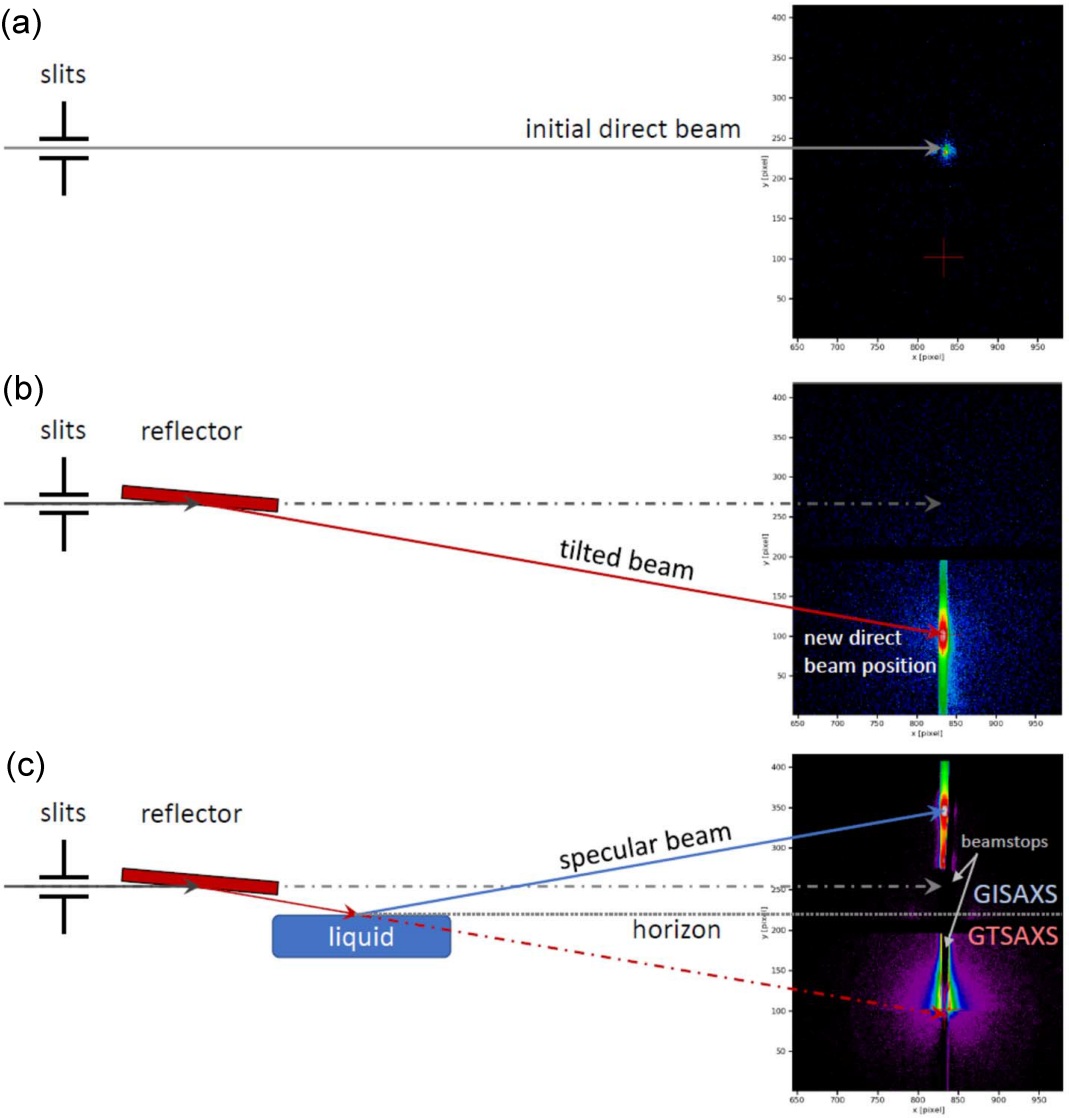
The geometry of the three different steps of the beam tilting procedure with the beam axes, main reflecting blocks and corresponding scattering image at the detector: (*a*) initial direct beam, (*b*) beam tilted by the reflector, and (*c*) transmitted and reflected beam from the liquid interface.

After assembling the main nodes of the reflector circuit for the GIUSAXS experiment, it is necessary to adjust all its nodes relative to the X-ray beam on each of them. The alignment procedure for the GIUSAXS setup involves the following key steps (see also Fig. 4 ▸; a detailed description is also provided in Section S3 of SI):

(1) Prepare the USAXS setup in transmission mode.

(2) Determine the direct beam position (DBP) at the detector.

(3) Align the reflector at zero angle using vertical (*Z*) and beam-axis tilt (CHI) scans. Set CHI = 0°.

(4) Tilt the reflector to an angle α_i_r_ = 0.2–0.4°, measure the distance *L*, and define the specular beam position (SBP) according to α_i_r_. Perform *Z*-alignment at α_i_r_ and set CHI = α_i_r_.

(5) Align the sample trough horizontally using a water balance and adjusting the CHI/PHI motors.

(6) Set the desired angle α_i_r_ = α_i_s_/2, and perform *Z*-alignment of the sample.

(7) Move to the maximum intensity position of the *Z*-scan of reflected beam on the detector.

(8) Repeat *Z*-alignment of the sample for each new angle α_i_r_ = α_i_s_/2.

(9) Check the liquid level regularly (every 20–30 min) with a *Z*-scan to compensate for evaporation. A lid with inlet/outlet windows placed above the trough is recommended.

Here, *L* is the reflector–detector distance (Fig. 1 ▸), *L*_1_ is the reflector–sample distance, *L*_2_ is the SDD.

The main geometries of USAXS beam tilting preparation are presented in Fig. 4 ▸. Initially, the horizontal focused X-ray beam for USAXS measurements, Fig. 4 ▸(*a*), will be tilted by the previously aligned reflector at the required angle α_i_r_ = α_i_s_/2. The reflected beam position at the detector will be a new direct beam position for each value of α_i_r_, Fig. 4 ▸(*b*). Upcoming to the reflected beam axis the liquid interface in the trough will mirror the specular beam after the alignment procedure of the sample, Fig. 4 ▸(*c*). As a result, a two-dimensional GISAXS pattern from the surface structure will be formed on the detector at incidence angles up to or near the critical angle of the liquid α_i_s_. It should be noted separately that beamstops (optional round or vertical rod beamstops) and absorbers must be installed in front of the detector to avoid damage during the adjustment process and when working in the area of critical reflection angles, Fig. 4 ▸(*c*).

The grazing-incidence transmitted signal of small-angle X-ray scattering (GTSAXS) is intrinsic for such an experiment geometry (Lu *et al.*, 2013 ▸). The surface horizon position at the 2D diffraction image can be estimated as
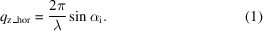
All the area above the surface horizon is a classical GISAXS signal, described by the distorted wave Born approximation (Sinha *et al.*, 1988 ▸), Fig. 4 ▸(*c*). The space below *q*_z_hor_ refers to the GTSAXS, which can be formed at an incident angle larger than the critical angle of the substrate and analyzed within the standard Born approximation (Lu *et al.*, 2013 ▸). The presence of (i) a sufficiently high-energy X-ray beam focused on the sample, incident at an angle greater than the critical angle, (ii) a low-absorbing substrate (liquid) and (iii) the possibility of scattering through the far edge of the sample (the rising meniscus of the liquid) are very suitable for GTUSAXS experiments and is one of the main advantages of the proposed geometry for the beam tilt USAXS experiment. The 2D images of the direct, tilted from the mirror and reflected from the liquid interface and X-ray beam are shown in Fig. S8 (SI). A summary of the key experimental parameters, including the incident angle range, corresponding *q*-resolution, beam separation at the sample, and usable energy range, is presented in Table S4 (SI). These specifications reflect the typical geometry and performance characteristics of the proposed GIUSAXS setup and are relevant for optimizing alignment and data collection conditions.

### 3.3. Alternative alignment approach using hexapod translation for the sample height correction

As an alternative to the standard vertical alignment of the sample, an additional approach can be considered using the full capabilities of the hexapod stage that holds the reflector. In this method, the hexapod is used not only for tilting the reflector to control the incidence angle but also for translating it along the X-ray beam direction. This allows the reflected beam to land on the same position on the sample surface as the incidence angle changes or as the liquid level drops due to evaporation, without requiring adjustment of the sample height.

While this beam-tracking method is conceptually feasible and can be implemented with high-precision hexapod systems, it introduces certain limitations. Although the beam footprint on the sample can be restored, the specularly reflected beam on the detector shifts vertically because of the altered reflection geometry. This effect can be negligible for small vertical displacements, but may become significant depending on the detector pixel size, SDD, foot-print beam size and length of the reflector, the amount of liquid level change, and would require additional correction of the detector position or beam axis during data acquisition or analysis. In addition, the displacement of the reflector towards the *X*-axis encounters a technical limitation of the hexapod translation in this direction (usually ∼25 mm), in addition to changing the reflector–sample distance *L*_1_, Fig. 1 ▸. This effect is illustrated in Section S4, Fig. S5 (SI), which compares the aligned, shifted and beam-realigned positions.

In addition, this beam-tracking strategy requires additional coordination and mechanical motion, potentially increasing complexity for non-expert users at synchrotron facilities. For these reasons, and in favor of robustness and ease of use, the standard alignment method based on adjusting the sample height (*Z*-position) was chosen as the default operating mode in our implementation. Nevertheless, the hexapod-based beam stabilization concept remains promising, particularly for future setups with automated control and real-time feedback.

### 3.4. Sample description and discussion

**Figure 5:**
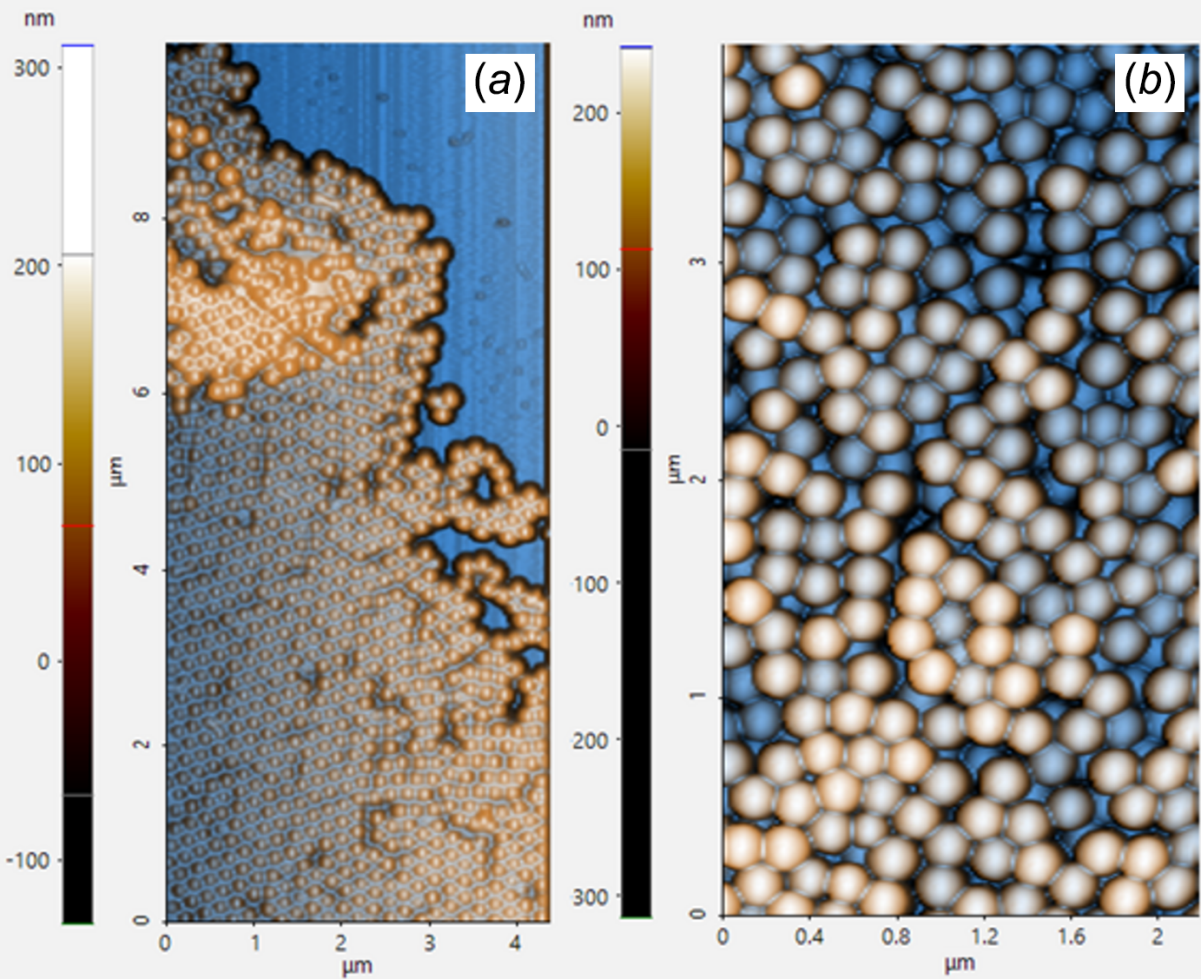
AFM 3D image of the PPS colloid agglomerate, transferred from the air/water interface after the X-ray experiment.

A colloidal dispersion of polystyrene spherical nanoparticles with diameter *D* = 197 ± 3 nm (PPS) was chosen as a test sample system for the study of the X-ray beam slope scheme for GIUSAXS on the liquid surface. To create the colloid layer at the liquid interface the 9 ml dispersion of polystyrene colloids (PPS-0.2, polystyrene microparticles, 25 mg ml^−1^; KISKER BIOTECH GmbH) was mixed with 1 ml of methanol (≥99.8%, Sigma-Aldrich) for spreading procedure during the evaporation of the methanol. The 60 µl of colloid/methanol mixture was spread on an ultrapure water surface in a Teflon trough (70 mm × 90 mm × 5 mm) using a glass slide at a shallow angle for colloid layer formation at the air/water interface, Fig. S7 (SI). The Teflon handle barrier was moved parallelly to the X-ray axis from one of the sides to collect the floating colloids at the interface for the dense layer creation. A 3D AFM image of the transferred and dried agglomerate of PPS colloid spheres is shown in Fig. S9(*a*) (SI). According to the height profile [Fig. S9(*b*), (SI)], the average diameter of the dried spheres is around 200 nm, which corresponds to the parameters of the producer (KISKER BIOTECH GmbH). Examples of the mono- and multilayer areas of the PPS assembly, transferred on the silicon wafer, are shown in Figs. 5 ▸(*a*) and 5(*b*), respectively. One or a few layers of colloid were found on the surface of the silicon substrate after drying the transferred film. The AFM experiment was performed using an NX10 atomic force microscope (Park Systems, Korea).

**Figure 6:**
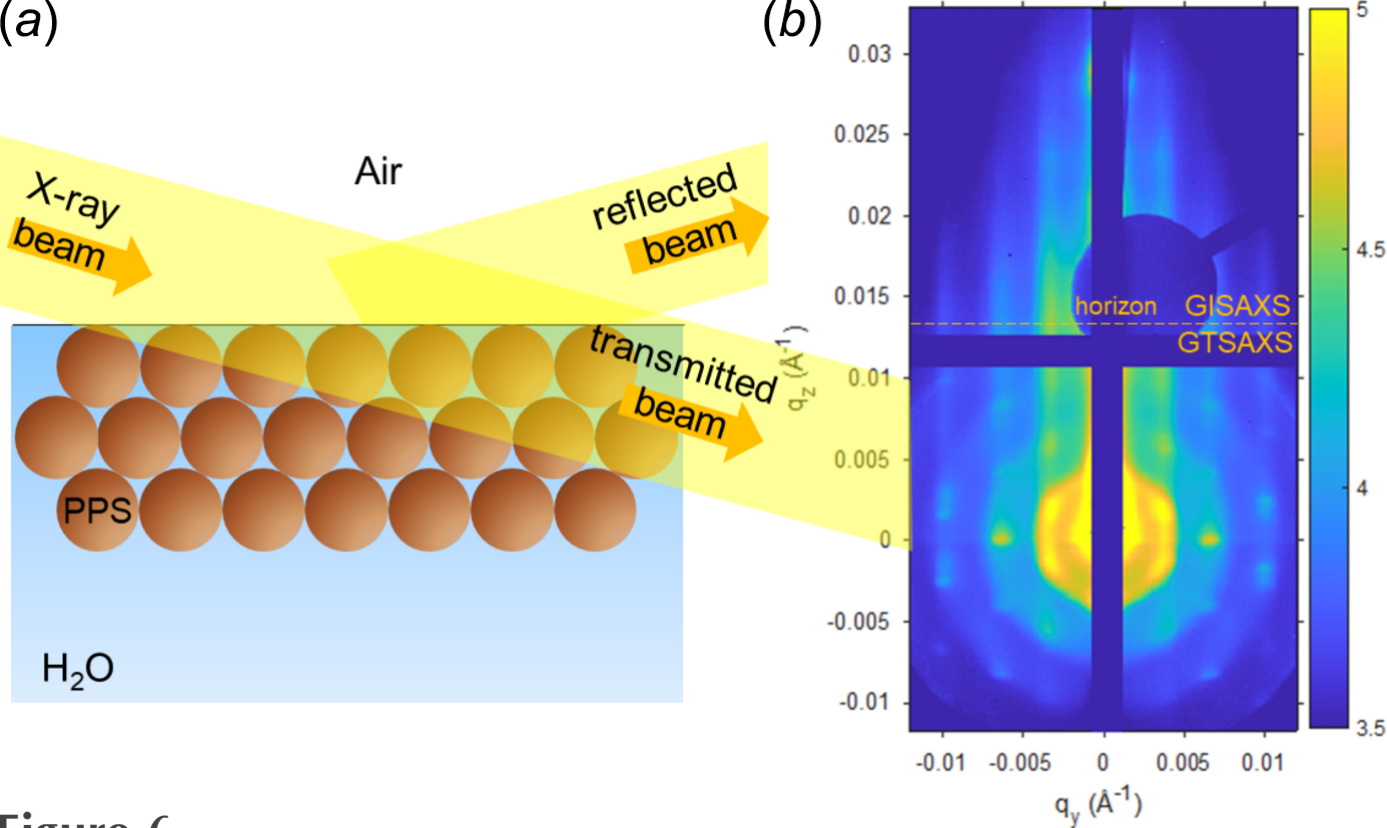
The PPS assembly under the air/water interface model and incident/reflected and transmitted X-ray beam (*a*), and 2D GISAXS/GTSAXS diffraction image (*b*)

When the focused X-ray beam (11.83 keV) was incident under angle α_i_s_ = 0.13° and subsequently reflected from the colloid layer at the air/water interface in the low-angle region, a two-dimensional diffraction pattern was formed on the detector area, Fig. 6 ▸. Considering the critical angle of the water α_c_H2O_ = 0.104° (*q*_z_ = 0.0109 Å^−1^), the penetration depth of the X-ray beam Λ ≃ 4.7 µm and the surface horizon position *q*_z_hor_ = 0.0136 Å^−1^ [equation (1)], the top and bottom part of the image can be interpreted according to two different conceptions. Calculated according to equation (S9) and beam width, the footprint area at the air/water interface at α__i_ = 0.13° was approximately 11 mm × 0.035 mm.

**Figure 7:**
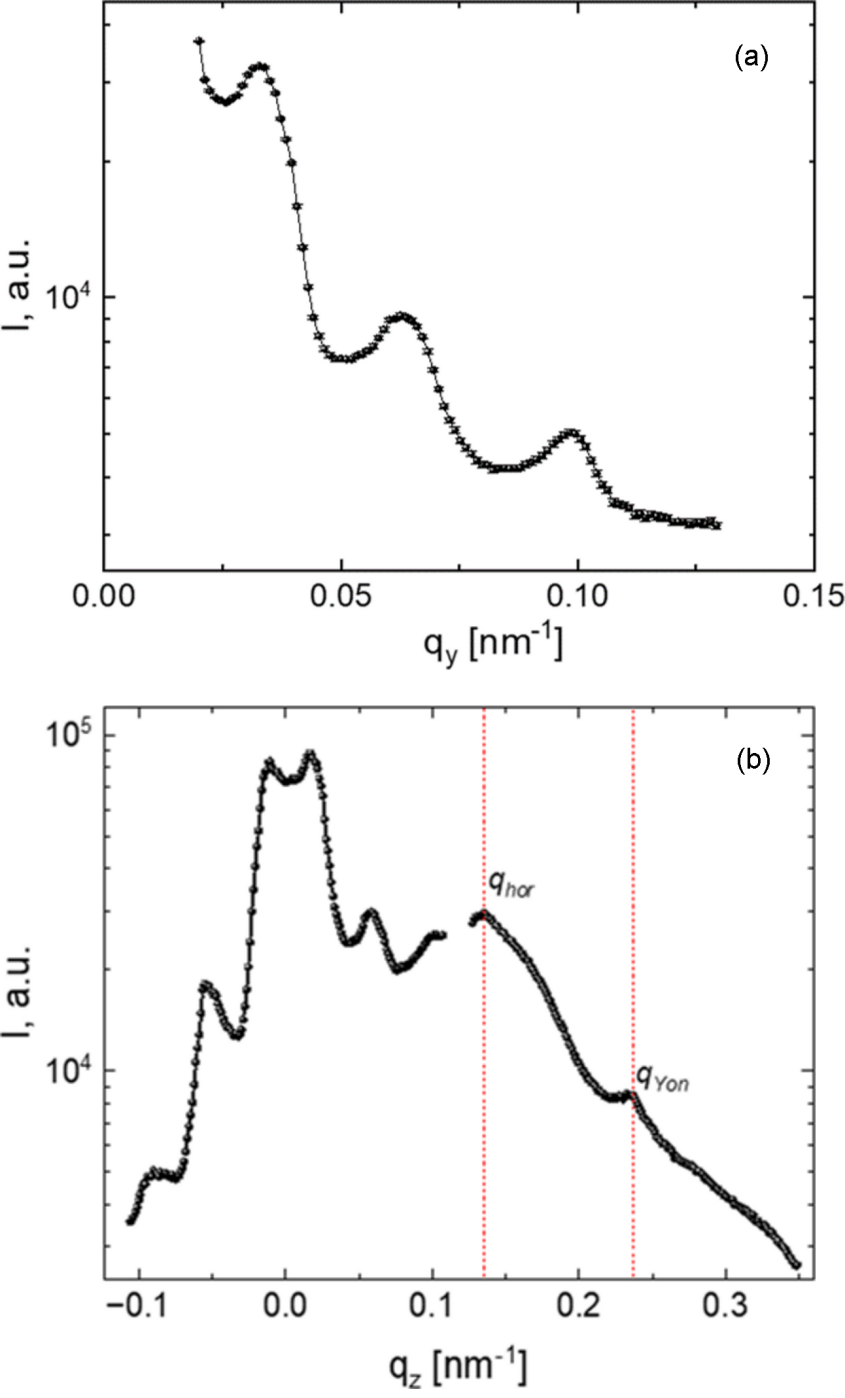
(*a*) Horizontal line cut *I*(*q*_y_) at *q*_z_ = 0.145 nm^−1^. (*b*) Vertical line cut *I*(*q*_z_) at *q*_y_ = 0.0335 nm^−1^.

The area at *q*_z_ > *q*_z_hor_ belongs to the GISAXS signal. The strongest peaks along *q*_y_ are the pseudo-Bragg peaks of a paracrystalline lattice. The structure factor dominates scattering along *q*_y_. A line horizontal cut *I*(*q*_y_) at the most intensive area above the horizon at *q*_z_ = 0.145 nm^−1^ [Fig. 7 ▸(*a*)] demonstrates the periodical peaks with the positions 0.0335 ± 2, 0.0635 ± 2 and 0.0975 ± 3 nm^−1^, that correspond to the position of first, second and fourth peaks of canonical 2D hexagonal lattice positions with corresponding ratios of 1, 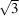 and 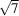, respectively. The actual position of the first-order peaks must be recalculated considering the center of mass shifting of the first-order peak according to the Fourier transform from spherical particles, as *q* = 0.92 × (2π/*d*), where *d* is an interplane distance. Considering the hexagonal structure of PPS packing of the top layer, the calculated averaged diameter of the PPS is *D* = 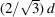 = 198 ± 2 nm, which corresponds to the initial size of the PPS. The results of the GIUSAXS simulation from the 2D layer of spheres above and below the interface are given below.

The area at *q*_z_ < *q*_z_hor_ belongs to the GTSAXS signal, Fig. 6 ▸. We may see a diffraction scattering pattern with clear hexagonally ordered peaks without diffuse scattering, which is an attribute of a three-dimensional hexagonally packed PPS crystal with the *C* axis oriented perpendicular to the surface of the water interface. The axis of the transmitted beam is directed perpendicular to the thickness of the detected section of the crystal from several PPS layers. Presumably, the formation of a three-dimensional hexagonal PPS structure from a monolayer under the interface occurs under the action of the directed movement of the barrier parallel to the beam axis.

Fig. 7 ▸(*a*) shows a section at *I*(*q*_z_) along the rod (10*l*) at *q*_y_ = 0.0335 nm^−1^ with the presence of modulated interference maxima along the *l* direction, covering the GISAXS and GTSAXS regions. The fitted positions of all the main peaks (−0.08912, −0.04915, −0.00991, 0.01632, 0.05559, 0.095, 0.1335 and 0.2342 nm^−1^) are presented in Fig. S10(*b*). The positions of the peaks at *q*_hor_ = 0.1335 nm^−1^ and *q*_Yon_ = 0.2342 nm^−1^ correspond to the calculated positions of the horizon, equation (1), and the Yoneda peak (α_f_ + α_c_). The average distance between adjacent peaks in the vertical *q*_z_ cut outside the first-order peaks is about 0.0393 ± 1 nm^−1^. The particle diameter calculated considering the correction for the shift of the peaks’ center of mass and the coefficient 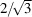 will give a value of 200 nm. This is the shortest distance of the 100 direction of hexagonal packing of the spheres and can be defined for both face-centered cubic or hexagonal closed-packed kinds of cell.

The positions of the first-order peaks near the direct (transmitted) beam are correctly analyzed by constructing the radial intensity distribution. The radial position of peaks of different orders was analyzed when performing individual narrow cake cuts. The positions of the main peaks on each section are 0.03596, 0.06447, 0.09619, 0.1013, 0.122 and 0.1332 nm^−1^ and also correspond to the scattering peaks from the hexagonal structure. Theoretical sets of Bragg reflections of the face-centered cubic structure correspond to the positions of the experimental peaks in the region of the transmitted direct beam. Some twinning of peaks in the direction of 111 long-range orders indicates that the boundary of the neighboring crystal domain with slightly different plane orientation falls into the beam illumination region. A more detailed analysis of the structure of the test sample is beyond the scope of the main topic of this article.

To evaluate the depth-dependent contributions to the GIUSAXS pattern of PPS nanoparticles self-assembled at the air/water interface, we performed representative *IsGISAXS* simulations (Fig. S12 of SI). These simulations compare scattering from PPS layers floating on top of the interface versus that below it, revealing a strong contrast in intensity distribution and angular symmetry. While particles at the surface show SAXS-like radial patterns with dominant direct scattering, particles below the interface produce weaker, rod-like features due to reflection/refraction at the water boundary. The results suggest that the majority of PPS particles in our experimental system are located slightly below the air/water interface. A detailed simulation description is provided in Section S4 of SI.

### 3.5. Depth-resolved GIUSAXS simulations of PPS layers

**Figure 8:**
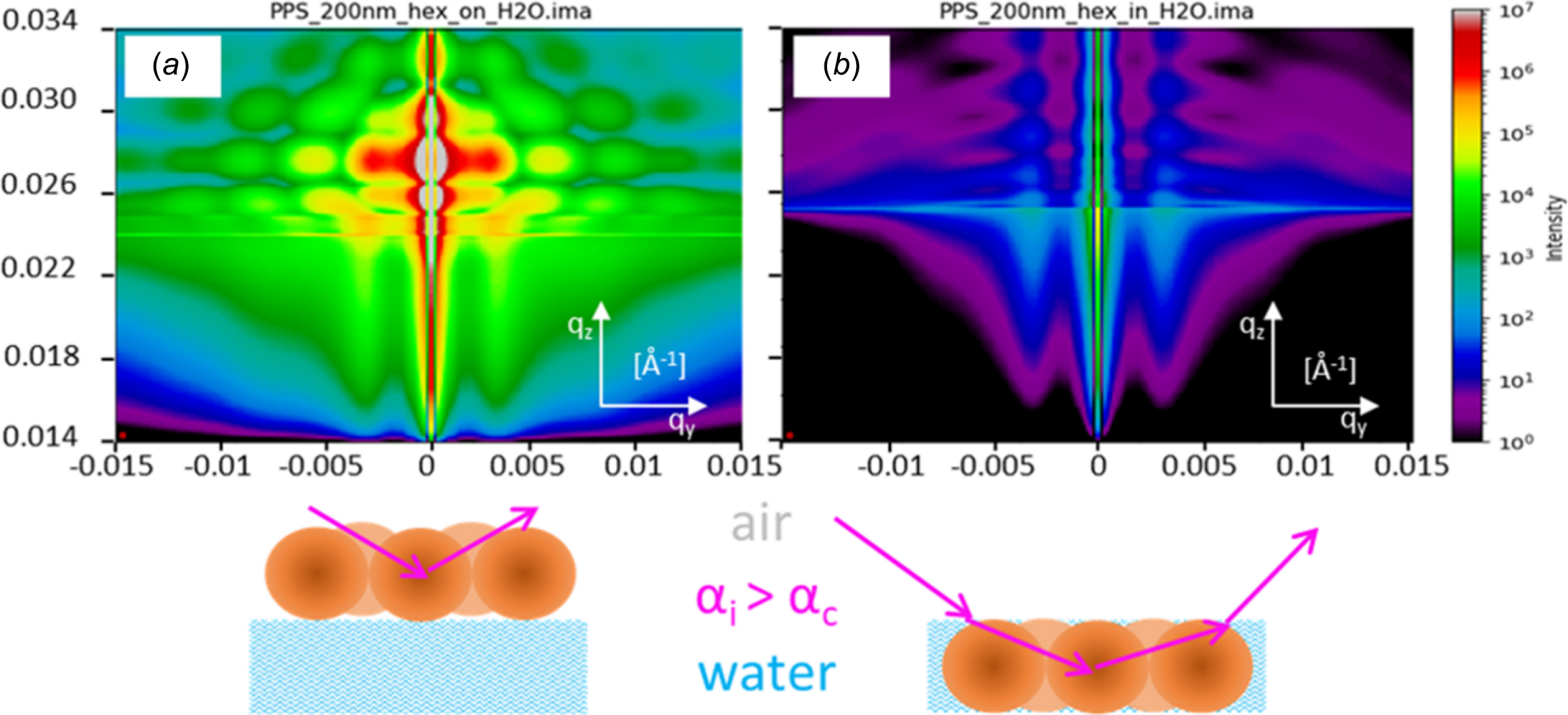
*IsGISAXS* simulation of representative GIUSAXS scattering patterns of 200 nm polystyrene nanoparticles self-assembled in a 2D-hexagonal lattice directly floating (*a*) on top and (*b*) below the air/water interface. The corresponding scattering intensity, scattering vectors as scale bars, and scattering geometry are indicated.

To visualize in-depth scattering contributions in the scattering pattern for self-assembly of polystyrene nanoparticles on top and below the air/water interface, representative simulations of scattering patterns are performed in the framework of GIUSAXS resolution considering the distorted wave Born approximation (DWBA) and a local monodisperse approximation for 200 nm spheres assembled in a 2D hexagonal lattice (Fig. 8 ▸). Simulations of full 2D scattering patterns were performed with *IsGISAXS* software V2.6 (Lazzari, 2002 ▸) with the same ultra-small angular resolution based on an input file containing all relevant information about the geometry and arrangement of nanoparticles (see the zip file in SI). The calculation of the interference function is based on a paracrystalline hexagonal 2D lattice (2ddlh) with a loss of long-range order. The Gaussian disorder parameter ω of the distance *D* = 200 nm was set to ω = 0.25*D*. The DWBA was used to calculate the form factor of full spheres with radii of 100 nm and a Gaussian distribution using σ/*R* = 0.5. For the PPS layers on top of H_2_O as substrate of depth 0 nm and PPS layers below H_2_O, a depth of 200 nm was considered (see Table S6 in SI).

Fig. 8 ▸ shows significant changes in the intensity distribution especially above the critical angles. For X-ray scattering of PPS floating on top of the air/water interface, the first channel of the DWBA, namely the direct X-ray scattering at the particle’s electron density, dominates the scattering pattern, whereas reflection events play a minor role. This can be seen alongside the SAXS-like signal around the specular reflection showing a hexagonal order similar to the GTSAXS signal in the presented data. However, for the scattering at the PPS layer floating below the air/water interface, the scattering contribution significantly changes yielding a dominance of reflection and refraction events at the air/water interface before and after the scattering at the PPS layers occurs. On the one hand, these events impact the overall intensity decreasing by approximately three orders of magnitude due to the loss of photons below the air/water interface and the reduced electron density contrast between water and PPS compared with PPS/air interfaces. On the other hand, the scattering geometry impacts the phenomenological appearance of the scattering pattern causing a break down in symmetry from radial intensity distributions along the specularly reflected peak to rather rod-like intensity modulations along *q*_z_. The form factors strongly smear out due to the predominating contributions of the refraction events, and the propagation of the incident and scattered waves start to feel the profile of the refraction index induced by the wafer substrate and the polystyrene particles themselves. Comparison with the key features observed in the experimental GIUSAXS data presented in Fig. 6 ▸ suggests that most of the scattering occurs at the PPS nanoparticles located below the air/water interface, indicating the formation of a PPS layer submerged beneath the liquid surface. The GTSAXS signal around the virtual direct beam position in turn averages all PPS in the illuminated volume regardless of the reflection and refraction events, and the Born approximation dominates the scattering with radial symmetry. However, *IsGISAXS* simulation software is not able to simulate GTSAXS signals or other possible X-ray pathways below the horizon. In addition, GTSAXS signals can overlap with the GISAXS signals at this low incident angle yielding some discrepancies between simulation and measurements. In other words, the consistency between the simulation and measurements is limited to the intensity distributions above the horizon at *q*_z_ = 0.0137 Å^−1^, *i.e.* the GISAXS signals only. Ultimately, a novel software kit able to simulate GISAXS and GTSAXS signals based on the same model would improve the comparison of simulation and measurement for future analysis.

## 4. Conclusions

This study demonstrates that while specialized synchrotron beamlines for *in situ* grazing-incidence experiments often suffer from limited angular resolution in the small- and ultra-small-angle range due to geometrical constraints, standard transmission-mode USAXS beamlines provide excellent resolution over a broad spatial range (up to several micrometres) but generally lack a flexible and cost-effective solution for beam tilting at liquid interfaces. To address this gap, we present a low-cost, modular beam-tilting scheme for enabling grazing-incidence USAXS (GIUSAXS) experiments on liquid surfaces. The system can be installed temporarily at the sample stage of most SAXS/USAXS facilities with minimal flux loss and without requiring beamline modification.

The approach uses polished solid reflectors with appropriate physical properties and low surface roughness to deflect the beam at small, well controlled angles. The maximum achievable incidence angle on the liquid surface is limited by approximately twice the critical angle of the reflector material, which is sufficient for the vast majority of GIUSAXS studies. We validated the setup through experiments on polystyrene nanoparticle colloids (194 nm in diameter) forming near-interface structures. The resulting GISAXS and GTSAXS patterns exhibit clearly distinguishable scattering features even in cases of low contrast across the liquid interface and across the critical angle.

Importantly, the proposed geometry is particularly advantageous for accessing the GTSAXS regime due to: (i) sufficient X-ray energy to probe beyond the critical angle, (ii) low absorption by the liquid substrate, and (iii) the ability to detect scattering from transmitted waves passing through the curved meniscus region. During experimental planning, trade-offs between incidence angle, beam energy, reflector dimensions, beam focus and the depth/position of the target structure relative to the interface should be carefully considered. Finally, the scheme is compatible with complementary *in situ* techniques such as GIWAXS and total reflection X-ray fluorescence, further broadening its utility for time-resolved and interface-sensitive studies.

## 6. Related literature

The following references, not cited in the main body of the paper, have been cited in the supporting information: Henke *et al.* (1993 ▸); Krieger & Petzold (1989 ▸); López-Flores *et al.* (2007 ▸); Schwartzkopf *et al.* (2013 ▸; Schwartzkopf *et al.* (2017 ▸).

## Supplementary Material

Sections S1 to S4 including Tables S1 to S6 and Figs. S1 to S11. DOI: 10.1107/S1600577525003431/ju5082sup1.pdf

The input files (*. imp), image files (*.ima) and output files (*.out) of the corresponding ISGISAXS simulation of PPS_200nm_hex_in_H2O or *_on_H2O. They are generally sharing all parameters in detail to enable reproduction of the simulation to everyone. In addition, there is a excel-file containing a sheet with q-calculation and second sheet with tabulated refractive indecies as basis for the simulation as it was requested. DOI: 10.1107/S1600577525003431/ju5082sup2.zip
