# Supplementary material for: Versatile X-ray reflector extension setup for grazing-incidence experiments at SAXS facilities for liquid surface study
Source: J Synchrotron Radiat. 2025 Jun 2;32(Pt 4):961–70. doi: 10.1107/S1600577525003431 (PMC12236259; doi:10.1107/S1600577525003431)
Supplement: Supplementary file 1 [file s-32-00961-sup1.pdf]

JOURNAL OF SYNCHROTRON RADIATION

**Volume 32 (2025)**

**Supporting information for article:**

# Versatile X-ray reflector extension setup for grazing-incidence experiments at SAXS facilities for liquid surface study

**Andrei Chumakov, Jan J. Rubeck and Matthias Schwartzkopf**

### S1. Theoretical Basis for X-ray Reflectivity and Incidence Angle Tuning

The main interactions of X-ray photons with matter are photoelectron absorption and X-ray scattering (elastic Rayleigh, inelastic Raman, and Compton scattering). Considering these interactions, along with Snell's law and Lorentz theory (where quasi-elastically bound atomic electrons oscillate due to the primary radiation), we analyze reflection and refraction of X-ray beams in the 6–25 keV energy range at the boundary between two media (Als-Nielsen & McMorrow, 2011).

The absorption of X-rays is related to the peculiarities of the interaction of photons with the electrons of the inner and outer shells, as well as with the atomic nuclei themselves. For photons with energies below 100 keV, the main contribution to the total attenuation is the photoelectric effect with a minor influence of X-ray scattering (Krieger & Petzold, 1989). It leads to substantial attenuation of the primary photons and emission of secondary photons with lower energies at absorption edges. This in turn will influence the number of photons involved in the scattering. We are mainly talking about the influence of K, L-I, L-II, and L_III edges for most elements and the energy range we are interested in. The remaining part of the interaction is a relatively minor part of the scattering process, which will be derived into the Rayleigh and Compton parts (Henke *et al.*, 1993). The Rayleigh scattering is predominant for energies below 90 keV, while the Compton part becomes dominant for energies of 90-200 keV (Als-Nielsen & McMorrow, 2011). Thus, when developing and using a reflector for GISAXS installation in the energy range of 6-25 keV, the photoelectronic effect and Rayleigh scattering should be the main influence.

An X-ray beam propagates in a homogeneous medium in a straight line. The beam will be deflected when it collides with the interface between the two media. In this case, its further propagation follows the following three rules: (i) The incident, reflected, and refracted beams will be in the same plane normal to the boundary plane; (ii) The glancing angles of the incident ($\alpha_1$) and reflected ($\alpha_1$ *) beams will be equal ($\alpha_1 = \alpha_1$ *), and (iii) The glancing angles of the incident and refracted beams follow Snell's law:

$$n_1 \cos \alpha_1 = n_2 \cos \alpha_2 , \quad \text{(S1)}$$

where $n_1$ and $n_2$ are the absolute refractive indices of the media 1 and 2, respectively.

Recall that vacuum is optically denser for X-rays than any material medium. The reflection index in vacuum $n_v = 1$, whereas the reflection index in media n is slightly less than 1 and is defined by:

$$n = 1 - \delta + i\beta, \quad \text{(S2)}$$

where $\delta$ is the real part, $i$ is the imaginary unit, $\beta$ is the imaginary component.

The imaginary component $\beta$ is responsible for the attenuation of the X-ray and is determined as:

$$\beta = \frac{\lambda}{4\pi}\left(\frac{\mu}{\rho}\right)\rho, \quad \text{(S3)}$$

where $\lambda$ is an X-ray wavelength, $\mu$ - attenuation length, and $\rho$ - density of the media.

The real part $\delta$ measures the deviation of the real component *n* = 1 - $\delta$ and can be written as

$$\delta = \frac{N_A}{2\pi} r_{el} \frac{Z}{A} \rho \lambda^2, \qquad \text{(S4)}$$

where $N_A$ is the Avogadro constant, $r_{el}$ is the classical electron radius, *Z* is the atomic number of the particular element, *A* is the atomic mass of the element (g/mol), $\rho$ is the physical density (g/cm$^3$), $\lambda$ is the wavelength of the primary X-ray beam (Å). It can be seen that the value of $\delta$ is individual for each of the elements and is of the order of $10^{-4}$ - $10^{-7}$ depending on the quantitative composition of the matter for the energy range from 6 to 30 keV and decreasing with increasing Å of the X-ray. The value of $\beta$ is usually one or two orders of magnitude smaller than $\delta$. From a practical point of view, when measuring samples, $\delta$ is responsible for photon scattering, whereas $\beta$ is responsible for radiation damage. Thus, to increase the scattering, lower beam energies can be chosen, whereas to reduce radiation damage to sensitive samples (soft matter), energies of the order of 20-30 keV are more suitable.

The phenomenon of total external reflection up to a certain angle of incidence results from the fact that a vacuum is an optically denser medium for X-rays than matter. This angle of incidence, after which the penetration of the beam into the sample begins, is called the critical scattering angle and in the case of the vacuum/matter interface is defined as:

$$\alpha_c \approx \sqrt{2\delta}, \qquad \text{(S5)}$$

Thus, the critical angle is directly related to the electron density (via δ) of the reflecting material and determines the angular limit for total external reflection. Typically, $\alpha_c$ for our chosen energy range lies between 0.04° and 0.6° and depends on the electronic strength of the reflecting material and the X-ray energy used. When the angle of incidence $\alpha_i$ decreases concerning $\alpha_c$, the reflectivity *R* increases up to 100%. Therefore, for the case $\alpha_i << \alpha_c$ the reflectivity R can be described as:

$$R \cong 1 - \sqrt{\frac{2}{\delta}\frac{\beta}{\delta}}\alpha_{inc}. \qquad \text{(S6)}$$

This parameter *R* is interesting for us to estimate the reflectivity of the mirror material for the chosen energy range.

When $\alpha_i$ increases relative to $\alpha_c$, the X-ray beam begins to penetrate the sample media. In this case, the penetration depth $\Lambda$ increases from a few nanometers to hundreds of microns and more in the case of a homogeneous medium, according to Eq:

$$\Lambda = \frac{\lambda}{4\pi}\sqrt{\frac{2}{\sqrt{(\alpha_i^2 - 2\delta)^2 + 4\beta^2} - (\alpha_i^2 - 2\delta)}} \qquad \text{(S7)}$$

and depends mainly on the $\beta$, which is proportional to the mass-absorption coefficient of the media. By increasing the angle of incidence $\alpha_i$ we can increase the depth $\Lambda$. This will allow us to selectively obtain information from a controlled depth of the sample relative to the surface, which is very important for many GISAXS/GIWAXS experiments, as well as in combination with TXRF.

To deflect the beam to a given angle, one of two types of elastically scattered photons is used in practice, such as diffraction and reflection at small angles in the grazing incident mode. Thus at the phenomenon of diffraction, there is a reflection of an incident wave from crystallographic planes and according to the Huygens principle also a generation of a monochromatic beam of secondary waves. This phenomenon is used in X-ray optics not only for monochromatization of the incident radiation but also for deflection of the beam to a significant calculated angle of several degrees and more, with the purpose of its subsequent use for the construction of a separate X-ray device or a separate measurement technique within the framework of a single facility. In particular, a single or dual crystal deflector is used to construct X-ray units with a controlled angle of incidence on a horizontal liquid surface. With the help of the deflector, it becomes possible to realize X-ray reflectivity (XRR) and GISAXS/GIWAXS techniques in a wide range of incidence angles.

In a single-crystal deflector, rotation of the tuned reflector crystal around the axis of the incident synchrotron beam sets the controlled inclination angle $\mu_{\max} = 2\theta$ of the Bragg angle of the used crystal to the investigated liquid surface (Smilgies *et al.*, 2005). The disadvantage of such a simple scheme of such an installation is the necessity to move the investigated liquid surface in space following the deflected beam. This creates additional technical difficulties and unnecessary mixing of the liquid sample due to inertia, which affects the measurement time and the quality of the collected data. Synchrotron facilities with a double crystal deflector avoid many such problems (Honkimäki *et al.*, 2006). In this case two single crystals with different crystallographic orientations, e.g. Ge(111)/Ge(220), Si(111)/Si(220), InSb(111)/InSb(220), or even Ge(333)/Ge(660) (Konovalov *et al.*, 2024), are used to create a tilted beam. The maximum slip angle $\mu_{\max} = 2(\theta_2$ and $\theta_1)$ in this case corresponds to twice the difference of the Bragg angles $\theta_2$ and $\theta_1$ of the second and first crystals, respectively, under the condition $\theta_2 > \theta_1$ (Murphy *et al.*, 2014). According to the two-crystal deflector scheme, changing the incidence angle $\mu$ no longer requires moving the sample. In addition, combining the two-crystal deflector with the sample node sampling tower and the diffractometer on a single platform allows us to significantly improve the stability and reproducibility of the entire setup (Konovalov *et al.*, 2024).

A small angle of incidence in the order of a few tenths of a degree can be realized with an X-ray mirror at the optical hutch at the beamline (Smilgies *et al.*, 2005; Konovalov *et al.*, 2024). However, this possibility of using a pre-integrated mirror is very rare and is rather an additional option. The disadvantage of using a mirror to tilt the beam is the limitation on the maximum achievable angle,

theoretically equal to several critical reflection angles of the material used. This technique creates difficulties for the implementation of the XRR technique but can be successfully used to obtain two-dimensional scattering patterns of GISAXS and GIWAXS. Thus, on the one hand, it is possible to deflect the beam while preserving the main part of the X-ray flux, and on the other hand, it is possible to use the tilted beam to study the liquid surface by changing the angle of incidence and its penetration depth into the sample. Understanding these interactions is crucial for optimizing GISAXS and GIUSAXS experiments, allowing precise control of penetration depth and sensitivity at liquid interfaces, as demonstrated in the proposed experimental setup.

## S2. Calculation and description

The above theoretical description will have two slightly different features concerning the reflector and the sample itself. It is known from theoretical calculations and practical measurements on real objects that the X-ray beam penetrates only a few nanometers deep into the material and slides along the reflecting surface until it reaches the critical angle ($\alpha_i < \alpha_c$). Its reflectivity in this case ranges from 100 % to 75-80 % depending on the reflecting material and the presence of absorption edges of the constituent elements. In practice, to achieve the highest reflection efficiency, the angle of incidence on the mirror $\alpha_{i\_r}$ is chosen to be about 80-85% of the value of $\alpha_c$ of the reflector material used for the selected X-ray beam energy. In our case, it gives the possibility to use the range of reflector inclination from 0 to 80-85% of the value $\alpha_{c\_r}$. Considering that the angle of incidence is equal to the angle of reflection, the chosen angle of reflector inclination $\alpha_{i\_r}$ relative to the beam axis will give the same angle of reflection. Thus, for the surface of a liquid sample, we will have a final angle of incidence equal to twice the reflector without significant loss of intensity of the beam falling on the sample relative to the initial one.

$$2\alpha_{i\_r} = \alpha_i \qquad \text{(S8)}$$

This means that even with comparable electron density of the reflector material and liquid surface, the experimenter has access to investigate both the air/liquid interface itself (up to $\alpha_c$ of the liquid) and to obtain a useful signal from a selected sample depth when $\alpha_c$ of the liquid is exceeded.

To investigate nanoobjects directly on the liquid interface, it is sufficient to choose an angle of incidence at the air/liquid interface equal to 80% of $\alpha_c$ of the liquid. In this case, the obtained scattering will contain information only of the nanoobjects directly floating on it, as well as ions with 2-3 nm of liquid thickness. Passing through the critical angle of the liquid interface, according to the formula (7), allows us to investigate the depth of the investigated nanoobjects relative to the surface, their structural parameters, and ordering by GIUSAXS/GIWAXS (and TXRF also) methods.

Table 1 presents theoretical calculations of various parameters for commonly used mirror materials in X-ray optics for the energy range from 5 to 30 keV. These materials are chemically inert and radiation stable under operating conditions in the air inside an experimental hutch for a pre-prepared monochromatic focused X-ray beam. A polished crystal of the desired size, or a polished commercially available lithography wafer, can be used as the mirror source for silicon or germanium. The silicon oxide mirror may be either the oxidized surface of a silicon single crystal or silicon wafer or polished quartz glass of the highest surface quality. Thin films of tabulated materials can be sputtered onto polished silicon by the widely used high-rate sputter deposition method to the conditions of a percolated closed coating with low roughness usually at thicknesses above 10 nm (Schwartzkopf *et al.*, 2013, 2017). For ease of evaluation, Table 1 also summarizes the calculated parameters for water as the most commonly encountered medium in air/liquid interface experiments. The given material density parameters were used for further calculations of the critical angle parameters for the critical mirror reflectivity at roughness = 0 nm (Henke *et al.*, 1993). The range of recommended X-ray energies for proposed mirrors is given in the third column, taking into account the maximal efficiency and absorption edges areas at the incident angles $\alpha_{i\_r} \leq 0.8\ \alpha_{c\_r}$. The subsequent columns are divided into three subgroups corresponding to the most frequently used X-ray energies, considering the recommended range for each material. Thus, the values of the critical angles $\alpha_c$ for each of the three energies allow us to estimate the value of the maximum recommended angle for the mirror $\alpha_{i\_r}$, as well as the maximum recommended angle of incidence on the sample surface $\alpha_{i\_s}$. The penultimate column gives an estimate of the average maximum ratio $\alpha_{i\_r}$ / $\alpha_{i\_s}$ of incidence angles for the air/water interface. From this we can see that for the most common types of experiment on water surfaces before and after $\alpha_c$ water, it is sufficient to use even such readily available materials as polished silicon or germanium.

The last column allows us to estimate the minimum transmission of the reflected beam (reflectivity) at the maximum values of angles $\alpha_{i\_r} = 0.8\ \alpha_{c\_r}$. These values will correspond to the maximum penetration depth of the beam into the liquid surface on the order of tens and hundreds of micrometers, see Table S1. However, this rule can be influenced by an increase in electron density when heavier elements are chosen and by the presence of absorption edges in the beam energy range of interest that can reduce the beam transmission by a few tens of percent (such as gold or platinum). For the reader's convenience, Support Information provides Table S2, which allows for estimating the X-ray absorption edges of the elements recommended for the mirror (http://skuld.bmsc.washington.edu/scatter/AS_periodic.html). At that, physical parameters of some elements allow approaching up to 90% of the $\alpha_c$ at the chosen energy without significant loss in intensity, slightly conceding in the size of the final maximum angle of reflection, but winning in transmission (Rh or Pd in comparison with Au and Pt). Commonly used ranges of incidence angles on the water surface in the neighborhood of $\alpha_c$ will allow to work in the range of reflectivity in the order

of 95-99.9% of the original beam. The reader can also make their estimates for other fluids with other densities for the air/liquid interface using the CXRO website (https://henke.lbl.gov/optical_constants/).

When calculating the size of the mirror to be used, it is important to consider the beam dimensions and the footprint of the beam on the mirror and the sample. It is assumed that a pre-trained monochromatic X-ray beam focused on a mounted mirror will have a minimum height dimension h of at most a few tens of microns. In this case, the footprint size $l_{fp}$ of the beam on the mirror will be determined by the incidence angle used on the mirror $\alpha_{i\_r} = \alpha_{i\_s}/2$ and can be calculated as (López-Flores *et al.*, 2007):

$$l_{fp} \approx \frac{h}{\sin(\alpha_i)} \qquad \text{(S9)}$$

Thus, if it is necessary to investigate the air/water interface at an angle less than the critical angle, $\alpha_{i\_s}$ for water will be 0.08° (80% of $\alpha_{c\_H2O}$ ) for a beam energy of 13 keV. In this case, the angle of incidence on the mirror should be $\alpha_{i\_r} = \alpha_{i\_s} / 2 = 0.04$ degrees, which for a vertical beam size of 25 µm would give a $l_{fp} \approx 35$ mm. The footprint at the air/water interface in this case would be half the size.

The transverse size of the mirror is usually much larger than the beam width (several tens of microns), being up to 10 mm or more. Such width of the mirror allows for adjusting its plane relative to the X-axis of the beam conveniently and quickly enough.

In addition to all the above-mentioned instrumental and physical aspects of the preparation, it is mandatory to consider all the special features of working with a liquid surface when carrying out any experiments at the air/liquid interface. Thus, attention should be paid to aspects such as gravity-capillary waves, surface curvature, active anti-vibration system, trough materials used, etc., as detailed in the literature (Pershan & Schlossman, 2012; Widom, 2004; Höfling & Dietrich, 2024; Konovalov *et al.*, 2022).

1) Discussing the possible limitations and optimization guidelines we may conclude, that while the GIUSAXS setup using a fixed-angle reflector offers a compact and transferable solution, several factors may affect optimal performance:

2) Beam divergence and background: The added air path between the mirror and sample can increase low-angle scattering from air molecules and mechanical components. This is especially relevant at low X-ray energies (6–10 keV) and when using long sample-detector distances (>2 m). To minimize this, the use of helium-filled or evacuated beam paths is strongly recommended.

3) Mechanical alignment sensitivity: Since the beam position shifts with each change in incidence angle ($\alpha_{i\_r}$), precise and stable positioning of both the mirror and the sample stage is essential. While

our setup uses simple manual adjustments, more advanced users may implement motorized Z-scanning for automated alignment.

4) Reflector surface quality: Any surface roughness or contamination of the mirror may lead to diffuse scattering. Polished Si or Ge wafers with sub-nanometer RMS roughness are preferred, and their cleanliness must be maintained throughout the experiment.

5) Absorption and flux loss: Although reflectivity is high (typically >95%) at incidence angles near 80–85% of $\alpha_c$, users should be aware of potential beam attenuation at higher angles or for materials with absorption edges in the chosen energy range.

These aspects should be carefully evaluated when designing experiments using this geometry, particularly in experiments requiring very high signal-to-noise ratios.

**Table S1** Theoretical calculations of various parameters for commonly used mirror materials in X-ray optics for the energy range from 5 to 30 keV. The parameters of critical and incident angles are shown for X-ray energies E = 8, 13, and 22 keV as an example

| Medium | ρ [g/cm$^3$] | $\Delta E_{eff}$ [keV] | $\alpha_{c\_r}$ [deg] | | | $0.8\alpha_{c\_r}$ [deg] | | | $\alpha_{i\_s\_max}$ [deg] | | | $\alpha_{i\_r}/\alpha_{i\_s}$ | $T_{min}$ [%] |
|---|---|---|---|---|---|---|---|---|---|---|---|---|---|
| E [keV] | | | 8 | 13 | 22 | 8 | 13 | 22 | 8 | 13 | 22 | | |
| **$H_2O$** | **1.00** | - | **0,154** | 0,095 | 0,056 | **0,12** | **0,08** | **0,04** | - | - | - | - | - |
| $SiO_2$ | 2.20 | 5.0 - 27.4 | 0,218 | 0,134 | 0,079 | 0,174 | 0,107 | 0,063 | 0,349 | 0,214 | 0,126 | 2,3 | 96,5 - 99,5 |
| **Si** | 2.33 | 5.0 - 27.0 | 0,224 | 0,138 | 0,081 | 0,179 | 0,110 | 0,065 | 0,358 | 0,221 | 0,130 | 2,3 | 94,1 - 99,1 |
| **Ge** | 5.32 | 5.0 - 11.0 | 0,311 | 0,192 | 0,116 | 0,249 | 0,154 | 0,093 | 0,498 | 0,307 | 0,186 | 3,3 | 90,1 - 92,2 |
| Mo* | 10.22 | 5.0 - 19.8 | 0,436 | 0,266 | 0.164* | 0,349 | 0,213 | 0.131* | 0,698 | 0,426 | 0.262* | 4,5 | 83,8 - 95,9* |
| Ag | 10.49 | 6.0 - 24.0 | 0,442 | 0,271 | 0,158 | 0,354 | 0,217 | 0,126 | 0,707 | 0,434 | 0,253 | 4,6 | 78,6 - 92,2 |
| Pd | 12.02 | 6.0 - 24.0 | 0,471 | 0,288 | 0,168 | 0,377 | 0,230 | 0,134 | 0,754 | 0,461 | 0,269 | 4,8 | 79,4 - 95,9 |
| **Rh** | 12.41 | 5.0 - 23.0 | 0,481 | 0,294 | 0,170 | 0,385 | 0,235 | 0,136 | 0,770 | 0,470 | 0,272 | 4,9 | 80,6 - 96,1 |
| Au** | 19.31 | 5.0 - 11.8 | 0,560 | - | - | 0,448 | - | - | 0,896 | - | - | 5,8 | 76,3 |
| Au** | | 15.0 - 24.0 | | 0,337 | 0,208 | - | 0,270 | 0,166 | - | 0,539 | 0,333 | 5,8 | 72,6 - 81,5 |
| Pt** | 21.45 | 5.0 - 11.4 | 0,588 | - | - | 0,470 | - | - | 0,941 | - | - | 6,1 | 77,1 |
| Pt** | | 14.0 - 24.0 | | 0,354 | 0,219 | - | 0,283 | 0,175 | - | 0,566 | 0,350 | 6,1 | 73,1 - 82,2 |

where:

$\Delta E_{eff}$ [keV] - range of recommended X-ray energies with maximal efficiency;

$\alpha_{c\_r}$ [deg] – the critical angle of the reflector’s material, calculated for E=8 keV

$0.8\alpha_{c\_r}$ [deg] - maximal recommended $\alpha_{i\_r} = 0.8\ \alpha_{c\_r}$, calculated for E=8 keV

$\alpha_{i\_s\_max}$ [deg] - the maximal incident angle of the X-ray beam at the liquid sample surface according to the maximal recommended $\alpha_{i\_r} = 0.8\ \alpha_{c\_r}$ for E=8 keV

$\alpha_{i\_r}/\alpha_{i\_s}$ - average ratio $\alpha_{i\_r}/\alpha_{i\_s}$ at maximum recommended $\alpha_{i\_r}$ for analyzed transmission range T (+/- 5%)

$T_{min}$ - minimal transmission range for mirror at the recommended E range according to $\alpha_{i\_r} \approx 0.8\alpha_{c\_r}$ [%]

* - Energy range, chosen according to the influence of the absorption of the K edge of the reflector material

** - Energy range, chosen according to the influence of the absorption of L-I - L-III edges of the reflector material

**Table S2** Theoretical calculations of penetration depth, incident angles ratio and transmission for commonly used mirror materials in X-ray optics for the energy range from 5 to 30 keV. The parameters of critical and incident angles are shown for X-ray energies E = 8, 13, and 22 keV as an example

| Medium | $\Delta E_{eff}$ [keV] | $\Lambda_{max}$ [µm] | | | $\alpha_{i\_r}/\alpha_{i\_s}$ | | | $T_{min}$ [%] | | | | | |
|---|---|---|---|---|---|---|---|---|---|---|---|---|---|
| | | | | | | | | $0.8\alpha_{c\_H2O}$ | $0.8\alpha_{c\_r}$ | $0.8\alpha_{c\_H2O}$ | $0.8\alpha_{c\_r}$ | $0.8\alpha_{c\_H2O}$ | $0.8\alpha_{c\_r}$ |
| *E* [keV] | | 8 | 13 | 22 | 8 | 13 | 22 | 8 | 8 | 13 | 13 | 22 | 22 |
| SiO2 | 5.0 - 27.4 | 5,5 | 15,8 | 51,2 | 2,3 | 2,3 | 2,3 | 98,3 | 96,5 | 99,2 | 98,6 | 99,8 | 99,5 |
| **Si** | 5.0 - 27.0 | 5,7 | 16,4 | 53,1 | 2,3 | 2,3 | 2,3 | 97,1 | 94,1 | 98,7 | 97,6 | 99,6 | 99,1 |
| **Ge** | 5.0 - 11.0 | 8,3 | 23,9 | 80,4 | 3,2 | 3,2 | 3,3 | 97,5 | 92,2 | - | - | 97,2 | 90,1 |
| Mo* | 5.0 - 19.8 | 11,9 | 34,1 | 110,3 | 4,5 | 4,5 | 4,5* | 96,3 | 83,8 | 98,2 | 92,6 | 99,2* | 95,9* |
| Ag | 6.0 - 24.0 | 12,1 | 34,7 | 112,1 | 4,6 | 4,6 | 4,5 | 95 | 78,6 | 97,6 | 92,2 | 99,2 | 80,8 |
| Pd | 6.0 - 24.0 | 12,9 | 37,1 | 119,1 | 4,9 | 4,9 | 4,8 | 95,5 | 79,4 | 97,8 | 90,7 | 99,2 | 95,9 |
| **Rh** | 5.0 - 23.0 | 13,2 | 37,8 | 120,9 | 5,0 | 5,0 | 4,9 | 95,9 | 80,6 | 98 | 91 | 99,3 | 96,1 |
| Au** | 5.0 - 11.8 | 15,5 | - | - | 5,8 | 5,7 | 5,9 | 95,6 | 76,3 | - | - | - | - |
| Au** | 15.0 - 24.0 | - | 43,6 | 149,0 | 5,8 | 5,7 | 5,9 | - | | 94,2** | 72,6** | 97 | 81,5 |
| Pt** | 5.0 - 11.4 | 16,3 | - | - | 6,1 | 6,0 | 6,3 | 95,9 | 77,1 | - | - | - | - |
| Pt** | 14.0 - 24.0 | - | 45,8 | 156,8 | 6,1 | 6,0 | 6,3 | - | | 94,6** | 73,1** | 97,3 | 82,2 |

where:

$\Delta E_{eff}$ [keV] - range of recommended X-ray energies with maximal efficiency;

$\Lambda_{max}$ - maximal penetration depth [um] of the X-ray beam in water at maximal $\alpha_{i\_s}$

$\alpha_{i\_r}/\alpha_{i\_s}$ - average ratio $\alpha_{i\_r}/\alpha_{i\_s}$ at maximum recommended $\alpha_{i\_r}$ for analyzed transmission range T (+/- 5%), calculated for the energies 8, 13, and 22 keV

$T_{min}$ - minimal transmission range [%] for the mirror at the recommended E range, calculated for the angles $\alpha_{i\_r} \approx 0.8\alpha_{c\_r}$ and $\alpha_{i\_H2O} \approx 0.8\alpha_{c\_H2O}$ for the estimation of the potential X-ray flux losing

* - Energy range, chosen according to the influence of the absorption of the K edge of the reflector material

** - Energy range, chosen according to the influence of the absorption of L-I - L-III edges of the reflector material

**Table S3** X-ray Absorption Edges of the elements recommended for the mirror. The elements are listed in order of increasing density. Bold font indicates absorption edges that fall within the 5-30 keV X-ray beam energy range selected for potential use.

| | Element Symbol | **Si** | **Ge** | **Mo** | **Ag** | **Pd** | **Rh** | **Au** | **Pt** |
|---|---|---|---|---|---|---|---|---|---|
| Edge (eV) | Element Number | 14 | 32 | 42 | 47 | 46 | 45 | 79 | 78 |
| K | | 1,839 | **11,103** | **20,000** | **25,514** | **24,350** | **23,220** | 80,725 | 78,395 |
| L-I | | - | 1,414 | 2,866 | 3,806 | 3,604 | 3,412 | **14,353** | **13,880** |
| L-II | | - | 1,248 | 2,625 | 3,524 | 3,330 | 3,146 | **13,734** | **13,273** |
| L-III | | - | 1,217 | 2,520 | 3,351 | 3,173 | 3,004 | **11,919** | **11,564** |
| M1 | | - | - | - | - | - | - | 3,425 | 3,296 |
| M2 | | - | - | - | - | - | - | 3,148 | 3,027 |
| M3 | | - | - | - | - | - | - | 2,743 | 2,645 |
| M4 | | - | - | - | - | - | - | 2,291 | 2,202 |
| M5 | | - | - | - | - | - | - | 2,206 | 2,122 |

**Table S4** Key Specifications of the GIUSAXS Setup

| **Specification** | **Value / Range** | **Comment** |
|---|---|---|
| Incident angle ($\alpha_{i\_s}$) | 0.04° – 0.6° | Covers angles below and above αc for typical liquids |
| q-range at 13 keV | 0.003 – 0.5 $nm^{-1}$ | Varies with αi and beam energy |
| q-resolution (Δq) | 0.0005 – 0.005 $nm^{-1}$ | Depends on beam size and sample-detector distance |
| Beam separation at sample | 0 – ~5 mm | Depends on tilt angle ($\alpha_{i\_r}$) and geometry |
| Energy range | 6 – 25 keV | Effective for most soft matter and liquid studies |
| Reflector angle ($\alpha_{i\_r}$) | 0.02° – 0.3° | Typically 80–85% of critical angle |
| Beam path ($L_1 + L_2$) | 5 – 30 m | Depends on beamline geometry and detector location |

**Table S5** Comparison of beam-tilting approaches for GISAXS on liquid surfaces

| **Feature / Setup** | **Double-Crystal Deflector (e.g., Arnold et al.)** | **Slit-Synchronized Scheme (JAC 2010)** | **This Work (Fixed Mirror)** |
|---|---|---|---|
| Beam footprint fixed during angle change | Yes | Yes | No |
| Angle adjustment method | Goniometer + 2-axis control | Synchronized slits | Manual sample Z-adjustment |
| Mechanical complexity | High | Moderate–High | Low |
| Alignment time | Long | Moderate | Short |
| Portability between beamlines | No (custom integration) | No | Yes |
| Cost (relative) | Very expensive | Expensive | Cheap |
| Vacuum compatibility | Yes | Yes | Yes |
| Compatible with high SDD / large q-range | Limited by space and complexity | Moderate | Yes |

**Aluminium-Vierkant-Rohre**

DIN 1748
Al Mg Si 0,5

| DESY-NR | Abmessungen | kg / m |
|---|---|---|
| 53 065 | 40 x 40 x 4 | 1,555 |
| 53 060 | 50 x 50 x 4 | 1,99 |

**Figure S1** Print-screen of the order of commercially available square section aluminum tube for producing the mirror holder, size 50 mm × 50 mm × 4 mm.

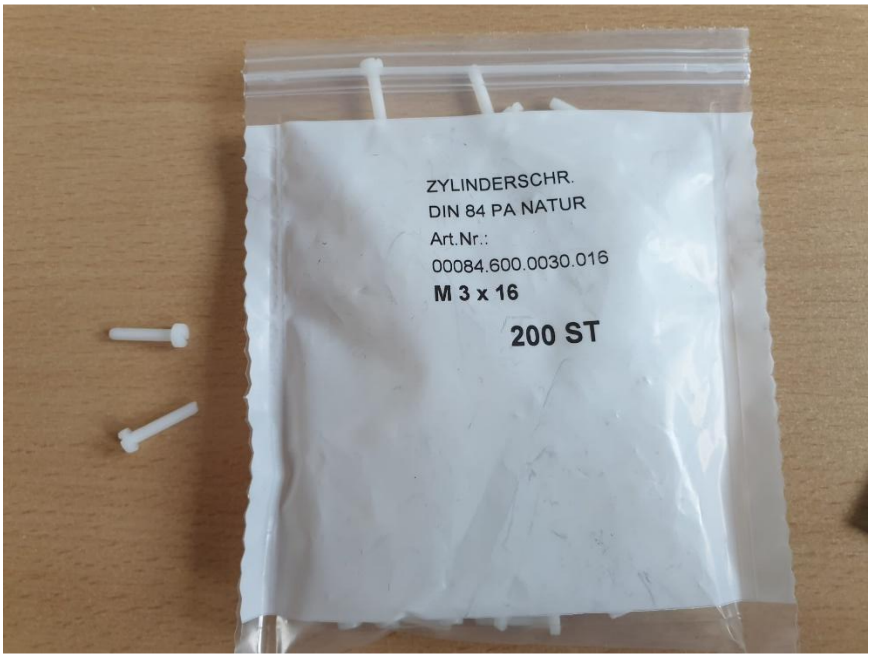


**Figure S2** Plastic bolts are used to fix the reflector according to the parameters of the Holder drawing

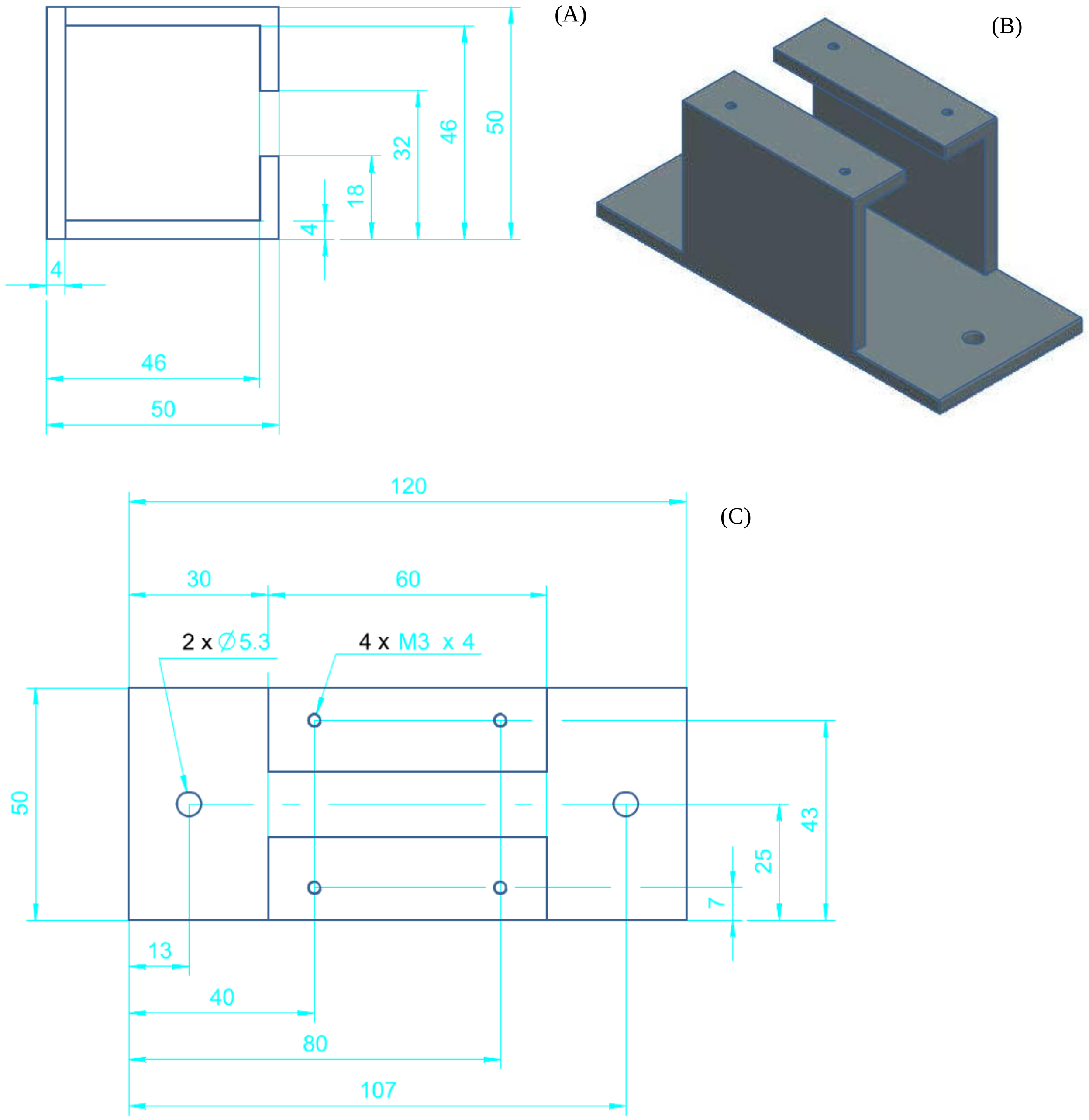


**Figure S3** The drawing of the base part of the reflector holder: (A) front view; (B) isometrical view; (C) top view

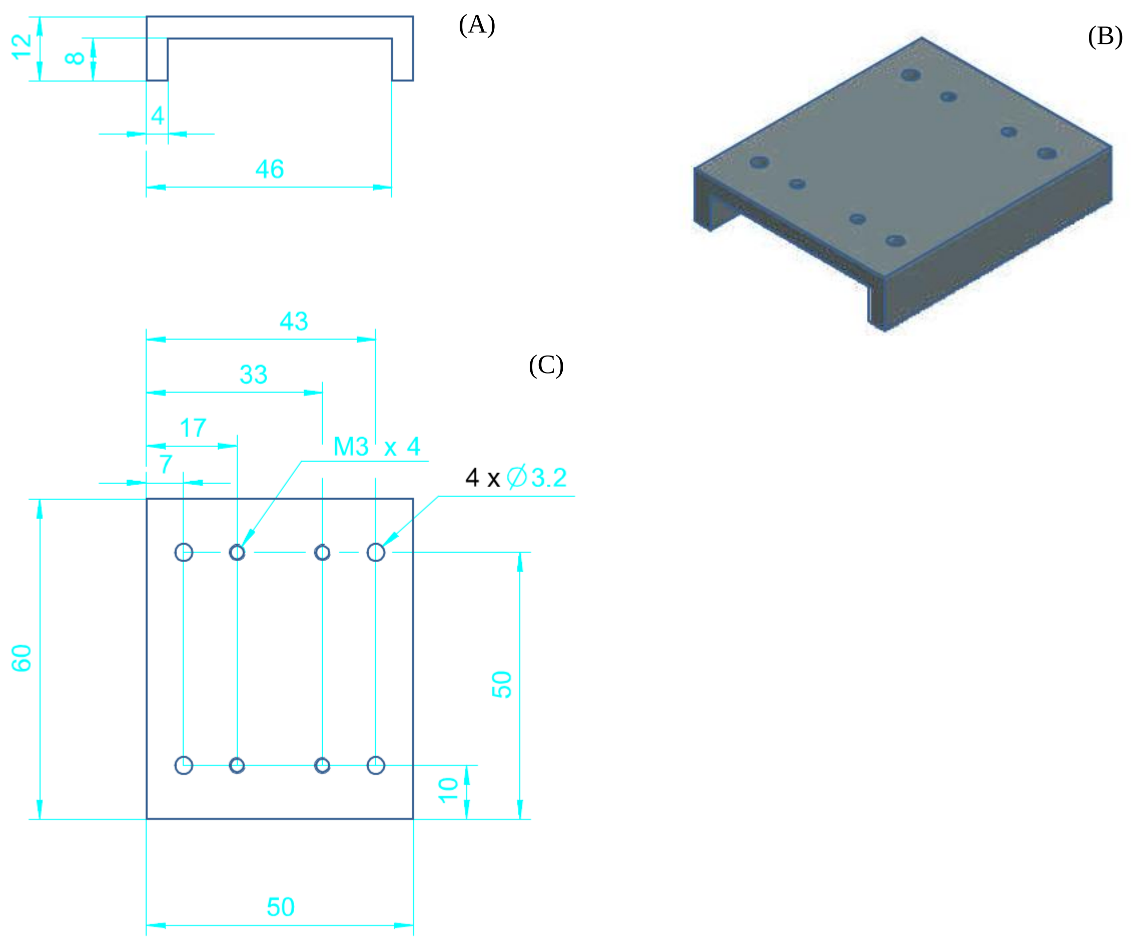


**Figure S4** The drawing of the clamping (top) part of the reflector holder: (A) isometrical view; (B) front view; (C) top view

**S3. Alignment procedure**

After assembling the main nodes of the reflector circuit for the GIUSAXS experiment, it is necessary to adjust all its nodes relative to the X-ray beam on each of them. The detailed description of alignment procedure is provided below:

1) Start form the prepared USAXS setup in transmission mode;

2) Determine the direct beam position (DBP) using a counter at the detector position.

3) Tuning of the reflector plane is performed similarly to the standard tuning of any flat sample for the GISAXS/GIWAXS experiment. For this purpose, a point detector (or a light-sensitive diode) is installed on the forward beam axis, or a region is selected on a 2D detector to count the forward beam photons. Successive alternating vertical (Z) scans and tilts along the beam axis (CHI) are then performed to adjust the reflector plane at zero angle (Figure 1). After reaching the intensity value in the sharp peak at the CHI angle swing equal to half of the maximum intensity of the step-functions curve from the Z adjustment, it is necessary to calibrate the current position at the maximum intensity CHI = 0. It is also necessary to check the zero position of the reflector plane in the transverse swing of the PHI angle so that in the end the X-ray beam reflected from the reflector is in the vertical plane of the direct beam axis.

4) The next stage of reflector angle adjustment is performed at any non-zero tilt angle. One of the options for adjustment is to bring the reflected beam from the reflector to the specified value pixel of the 2D detector. The position of the reflected beam is considered (Specular Beam Position, SBP [px]) relative to the center of the direct beam considering the specified tilt angle and calibrated distance from the vertical axis of the reflector to the GIUSAXS plane of the detector:

$$SBP = DBP \mp \frac{L \cdot \tan(2\alpha_{inc})}{PS} \qquad \text{(S10)}$$

where *DBP* - direct beam position in vertical direction [px], $L = (L_1 + L_2)$ - distance between the reflector and detector's position, $\alpha_i = \alpha_{i\_r}$ for the reflector alignment, *PS* - pixel size. Minus at the equation corresponds to the downward orientation of the normal of the reflector plane. The final adjustment step is to recalibrate the reflector angle to the calculated value. After this step, the user can deflect the reflector to any of the desired reflection angles.

5) After the liquid sample trough is installed, the trough is aligned horizontally using water balance and tilting CHI/PHI motors, and then the center of the trough is adjusted to determine the SDD ($L_2$ ). Depending on the purpose of the experiment and the type of liquid, the trough is filled with the set meniscus level.

6) Set the desired angle $\alpha_{i\_r} = \alpha_{i\_s}/2$, and perform Z-alignment of the sample. The center of the liquid sample is adjusted relative to the tilted X-ray beam by scanning the sample node in height (Z motor, Figure 1). Next, a plot is made of the change in the position of the reflected beam on the 2D detector

relative to the scanned Z motor, followed by the Z motor moving to the center position of the scanned peak.

7) Perform Z-alignment of the sample for each new angle $\alpha_{i\_r} = \alpha_{i\_s}/2$.

8) Check the liquid level regularly (every 20-30 min) with a Z-scan to compensate for evaporation. A lid with inlet/outlet windows may also be placed over the trough to minimize the evaporation of the liquid under investigation and to eliminate the influence of air currents in the experimental hutch.

The geometries of all main steps of USAXS beam tilting preparation are presented in Figure 4. Initially, the horizontal focused X-ray beam for USAXS measurements, Figure 4(A), will be tilted by the previously aligned reflector at the required angle $\alpha_{i\_r} = \alpha_{i\_s}/2$. The reflected beam position at the detector will be a new direct beam position for each value of $\alpha_{i\_r}$, Figure 4(B). Upcoming to the reflected beam axis the liquid interface in the trough will mirror the specular beam after the alignment procedure of the sample, Figure 4(C). As a result, a two-dimensional GISAXS pattern from the surface structure will be formed on the detector at incidence angles up to or near the critical angle of the liquid $\alpha_{i\_s}$. It should be noted separately that beamstops (optional round or vertical rod beamstops) and absorbers must be installed in front of the detector to avoid damage during the adjustment process and when working in the area of critical reflection angles, Figure 4(C).

**S4. Alternative alignment scheme using reflector translation along the X-ray beam direction**

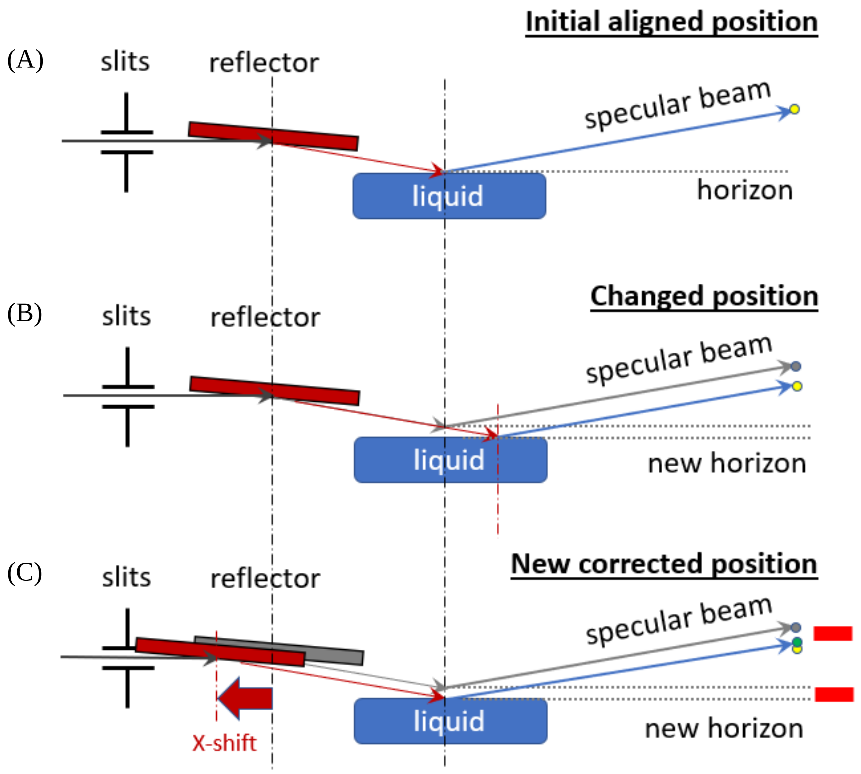


**Figure S5** Alternative alignment scheme using reflector translation along the X-ray beam direction.

The schematic illustrates three stages of beam position correction when the liquid level in the trough decreases during an experiment:

(A) Initial aligned position: the reflected beam hits the center of the liquid surface, and the specular beam is directed to its expected position on the detector.

(B) Changed position: due to evaporation, the liquid surface drops, and the reflected beam no longer hits the center of the sample, while the specular beam shifts downward on the detector.

(C) New corrected position: to restore the beam-sample alignment without moving the sample, the reflector is translated upstream along the beam axis (X-shift). The incident beam again hits the center of the liquid surface. However, the specular beam remains displaced on the detector compared to the initial geometry.

This scheme highlights a potential limitation of using only reflector translation to correct for evaporation-induced misalignment. While the beam position on the sample can be recovered, the angular geometry changes, leading to a shift in the specular beam position. This may affect data interpretation, particularly in GISAXS and GTSAXS measurements where precise angular registration is important. To avoid such complications, the main setup uses sample Z-adjustment as the standard method for maintaining alignment during liquid-level changes.

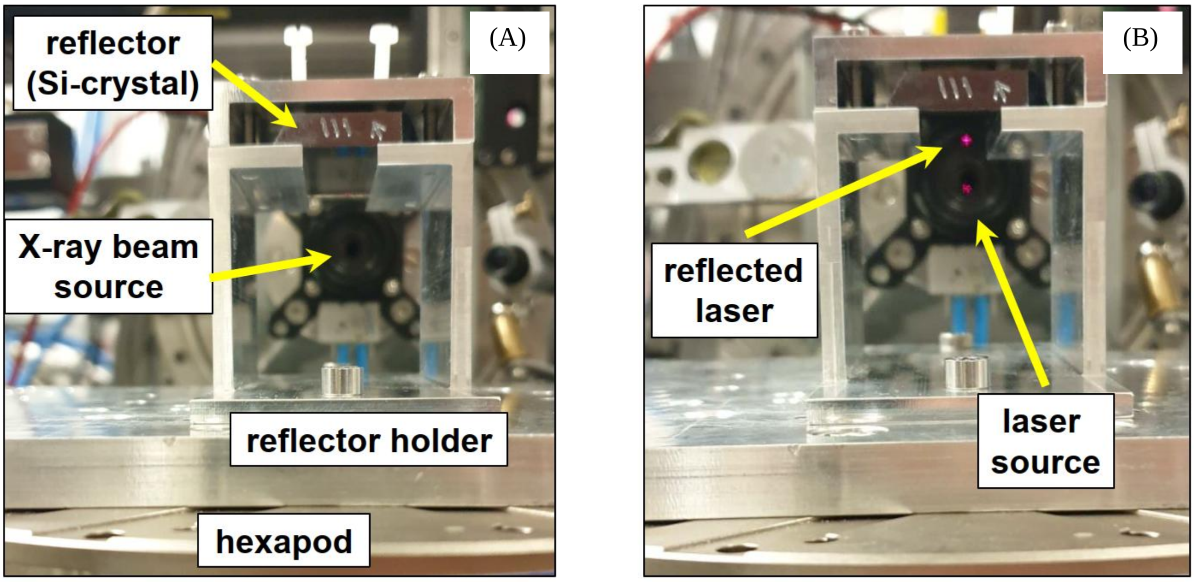


**Figure S6** (A) Silicon crystal surface mounted at the synchrotron station used as an X-ray beam reflector. (B) Demonstration of an X-ray beam-tuned silicon reflector surface. To visualize the position of the X-ray beam, a red laser was used whose axis coincides with the axis of the X-ray beam.

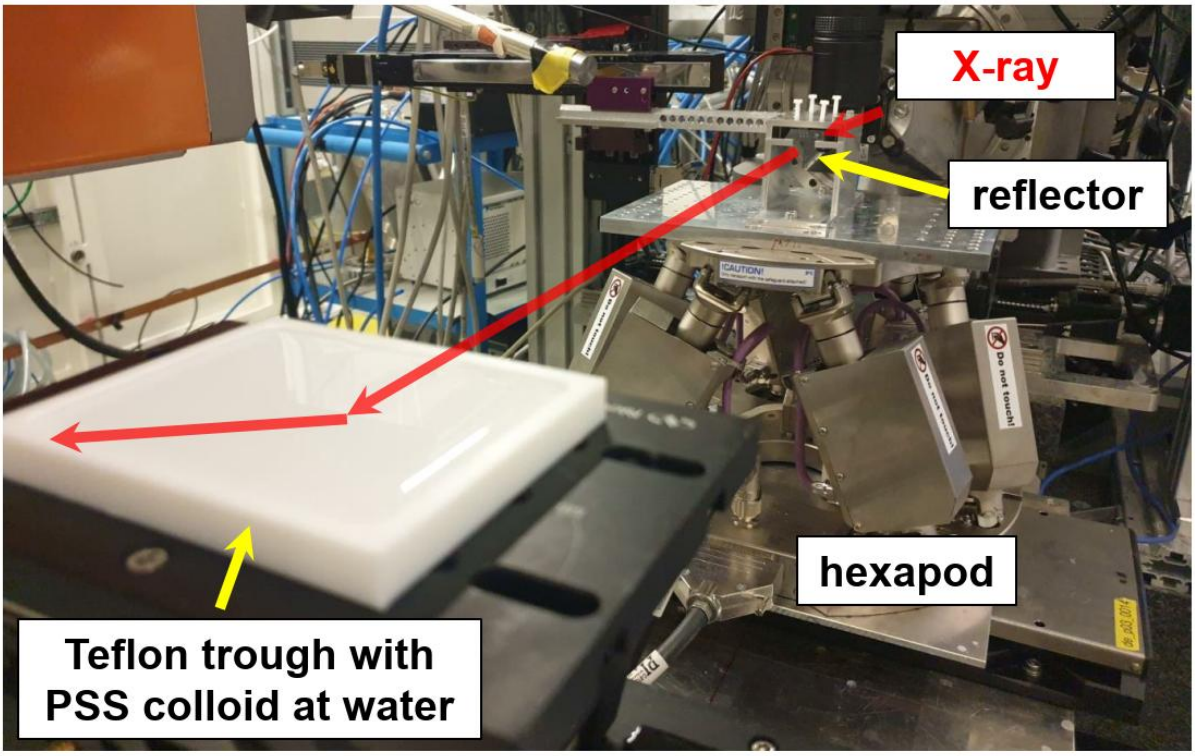


**Figure S7** Teflon trough with a test sample of polystyrene colloidal spheres (PSS) at the air/water interface during an X-ray experiment in the EH1 hutch of the P03 beamline

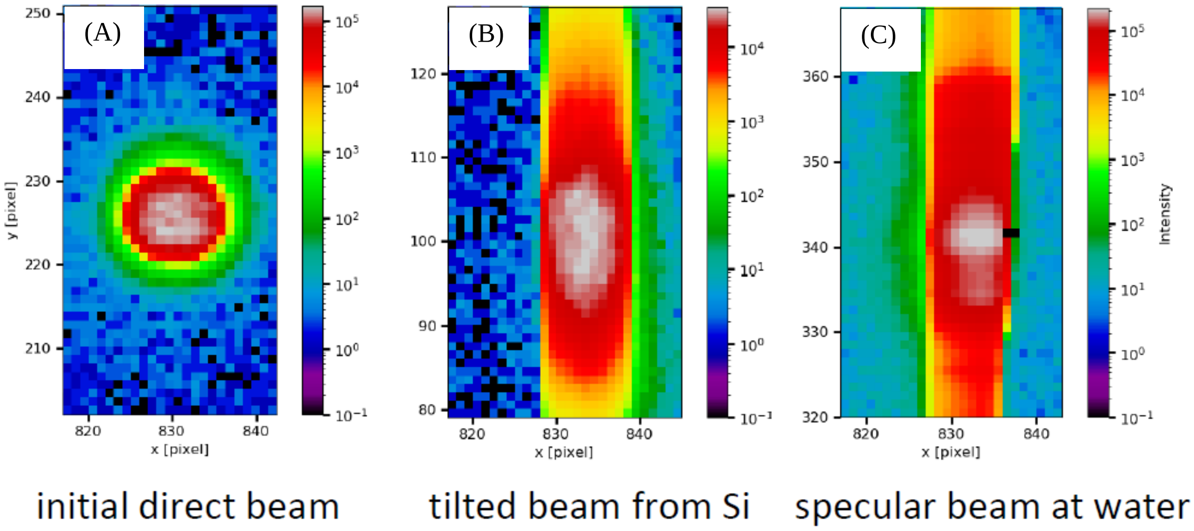


**Figure S8** 2D X-ray beam images for the (A) direct beam; (B) beam, reflected from the mirror; and (C) beam, reflected from the liquid (water) interface

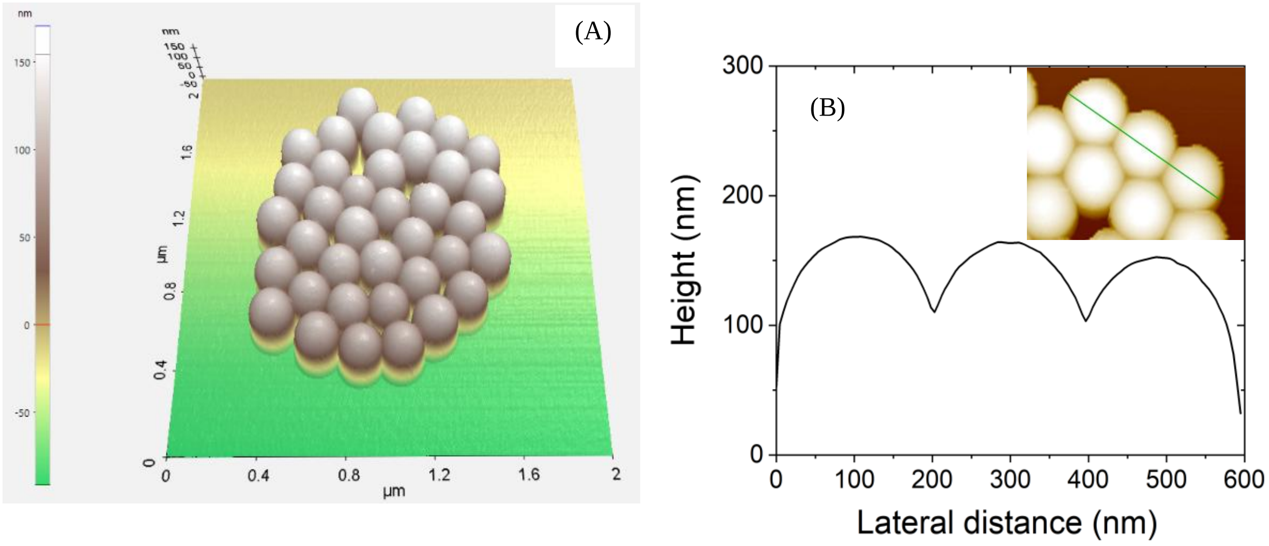


**Figure S9** (A) The AFM 3D image of the PSS colloid agglomerate, transferred from the air/water interface. (B) the AFM height profile of the dried agglomerate of polystyrene colloids (KISKER BIOTECH GmbH), transferred after the X-ray experiment from the air-liquid interface on the Si-wafer substrate. The insert figure demonstrates the cut line at the AFM image.

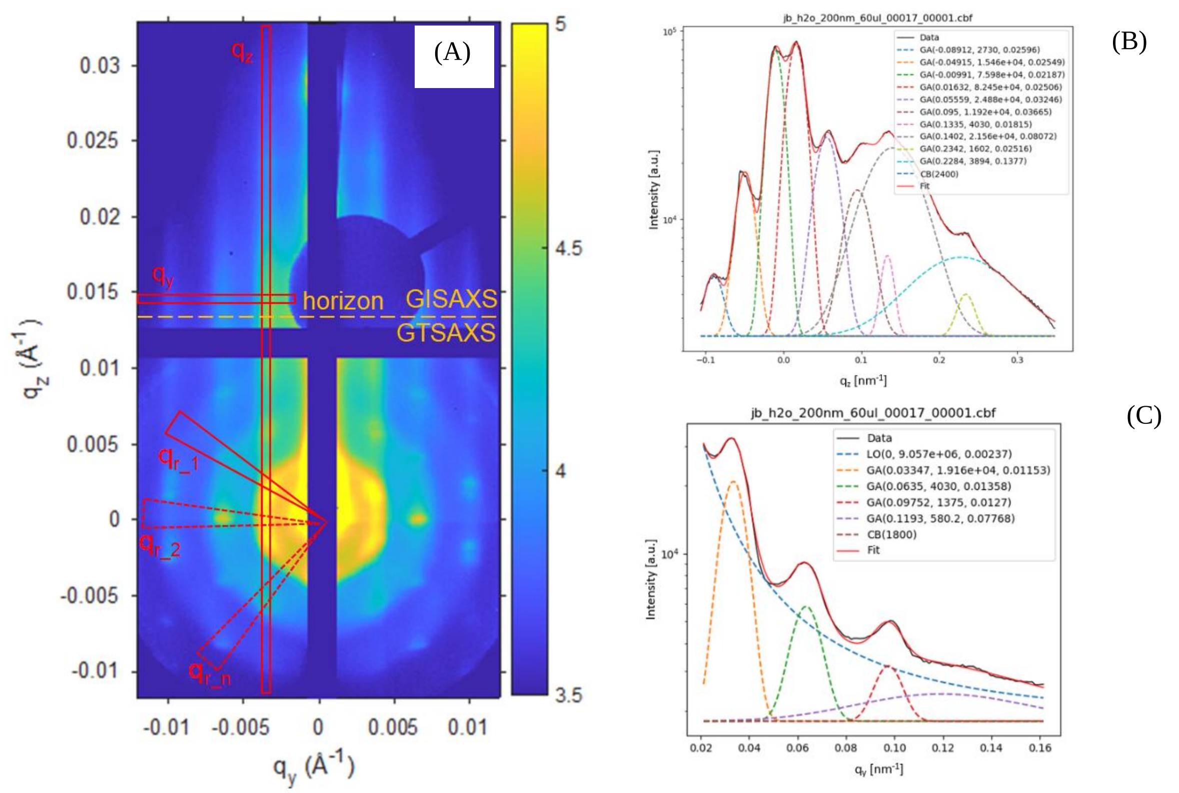


**Figure S10** 2D diffraction image of GISAXS/GTSAXS from PPS assembly under the air/water interface with the cut area for the subsequent analysis (A). The examples of the fit of horizontal line cut $I(q_y)$ at $q_z = 0.145\ nm^{-1}$ (B) and vertical line cut $I(q_z)$ at $q_y = 0.0335\ nm^{-1}$ (C)

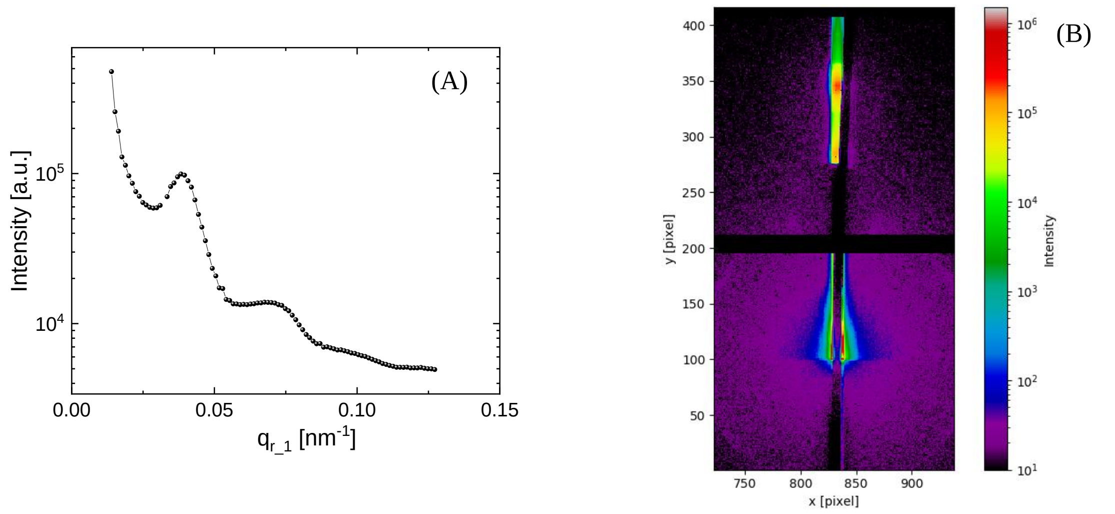


**Figure S11** (A) The examples of the radial line cut $I(q_{r\_1})$. The scattering pattern from pure water before adding PSS colloid (B).

**Table S6** Overview of main parameters of full 2D scattering patterns used for GISAXS simulations with IsGISAXS software V2.6 (Lazzari, 2002)

| Parameter | PPS on H2O | PPS in H2O |
|---|---|---|
| Framework | DWBA | DWBA_BURIED |
| Depth nm | 0 | 200 |
| Form Factor | full_sphere | full_sphere |
| Radius nm | 100 | 100 |
| R_distribution | Gaussian | Gaussian |
| SigmaR/R | 0.5 | 0.5 |
| Particle distribution | 2ddlh* | 2ddlh* |
| Distance [nm] | 200 | 200 |
| w (nm) | 50 | 50 |
| D_distribution | Gaussian | Gaussian |

Where „2ddlh" is 2D paracrystalline hexagonal lattice

**References SI:**

Als-Nielsen, J. & McMorrow, D. (2011). Elements of Modern X-ray Physics Wiley.
Henke, B. L., Gullikson, E. M. & Davis, J. C. (1993). *At. Data Nucl. Data Tables* **54**, 181–342.
Höfling, F. & Dietrich, S. (2024). *J. Chem. Phys.* **160**, 104107.
Honkimäki, V., Reichert, H., Okasinski, J. S. & Dosch, H. (2006). *J. Synchrotron Radiat.* **13**, 426–431.
Konovalov, O., Rein, V., Saedi, M., Groot, I. M. N., Renaud, G. & Jankowski, M. (2024). *J. Appl. Crystallogr.* **57**, 258–265.
Konovalov, O. V, Belova, V., La Porta, F., Saedi, M., Groot, I. M. N., Renaud, G., Snigireva, I., Snigirev, A., Voevodina, M., Shen, C., Sartori, A., Murphy, B. M. & Jankowski, M. (2022). *J. Synchrotron Radiat.* **29**, 711–720.
Krieger, H. & Petzold, W. (1989). Strahlenphysik, Dosimetrie und Strahlenschutz Wiesbaden: Vieweg+Teubner Verlag.
Lazzari, R. (2002). *J. Appl. Crystallogr.* **35**, 406–421.
López-Flores, V., Ansell, S., Bowron, D. T., Díaz-Moreno, S., Ramos, S. & Muñoz-Páez, A. (2007). *Rev. Sci. Instrum.* **78**, 13109.
Murphy, B. M., Greve, M., Runge, B., Koops, C. T., Elsen, A., Stettner, J., Seeck, O. H. & Magnussen, O. M. (2014). *J. Synchrotron Radiat.* **21**, 45–56.
Pershan, P. S. & Schlossman, M. (2012). Liquid Surfaces and Interfaces Cambridge University Press.
Schwartzkopf, M., Buffet, A., Körstgens, V., Metwalli, E., Schlage, K., Benecke, G., Perlich, J., Rawolle, M., Rothkirch, A., Heidmann, B., Herzog, G., Müller-Buschbaum, P., Röhlsberger, R., Gehrke, R., Stribeck, N. & Roth, S. V (2013). *Nanoscale* **5**, 5053.
Schwartzkopf, M., Hinz, A., Polonskyi, O., Strunskus, T., Löhrer, F. C., Körstgens, V., Müller-Buschbaum, P., Faupel, F. & Roth, S. V (2017). *ACS Appl. Mater. Interfaces* **9**, 5629–5637.
Smilgies, D.-M., Boudet, N., Struth, B. & Konovalov, O. (2005). *J. Synchrotron Radiat.* **12**, 329–339.
Widom, B. (2004). *Phys. Today* **57**, 66–67.
http://skuld.bmsc.washington.edu/scatter/AS_periodic.html

https://henke.lbl.gov/optical_constants/
